# Resting EEG correlates of neurodevelopment in a socioeconomically and linguistically diverse sample of toddlers: Wave 1 of the *Kia Tīmata Pai* best start New Zealand study

**DOI:** 10.1016/j.dcn.2023.101336

**Published:** 2023-12-26

**Authors:** Anne B. Arnett, Hayley Guiney, Tugce Bakir-Demir, Anita Trudgen, William Schierding, Vincent Reid, Justin O’Sullivan, Peter Gluckman, Elaine Reese, Richie Poulton

**Affiliations:** a Developmental Medicine, Boston Children’s Hospital, Boston, MA, USA; b Pediatrics, Harvard Medical School, Cambridge, MA, USA; c Department of Psychology, University of Otago, Dunedin, New Zealand; d Liggins Institute, University of Auckland, Auckland, New Zealand; e Department of Ophthalmology, University of Auckland, Auckland, New Zealand; f School of Psychology, University of Waikato, Hamilton, New Zealand

**Keywords:** Development, Language, Executive functioning, Temperament, Self-regulation, Pediatrics, EEG

## Abstract

Development of communication and self-regulation skills is fundamental to psychosocial maturation in childhood. The *Kia Tīmata Pai* Best Start (KTP) longitudinal study aims to promote these skills through interventions delivered at early childcare centers across New Zealand. In addition to evaluating effects of the interventions on behavioral and cognitive outcomes, the study utilizes electroencephalography (EEG) to characterize cortical development in a subsample of participating children. Here, we present results of the baseline resting EEG assessment with 193 children aged 15 to 33 months. We identified EEG correlates of individual differences in demographics, communication abilities, and temperament. We obtained communication and behavior ratings from multiple informants, and we applied contemporary analytic methods to the EEG data. Periodic spectral power adjusted for aperiodic activity was most closely associated with demographic, language, and behavioral measures. As in previous studies, gamma power was positively associated with verbal language. Alpha power was positively associated with effortful control. Nonverbal and verbal language measures showed distinct associations with EEG indices, as did the three temperament domains. Our results identified a number of candidate EEG measurements for use as longitudinal markers of optimal cortical development and response to interventions in the KTP cohort.

The first years of human life are marked by rapid neurocognitive and psychosocial growth. Among the most notable domains of early cognitive-behavioral development are language and self-regulation, both of which facilitate healthy relationships, learning, and emotion control. To date, the early neurobiological mechanisms for these behaviors are not well understood. Improved knowledge of neurodevelopmental processes promoting typical and neurodivergent psychosocial growth in very young children has the potential to inform delivery of precisely timed, targeted interventions to children who present with risk of developmental delays.

Verbal language development occurs rapidly in childhood, with children typically speaking single words around their first birthday, followed by combining 2–3 words into phrases by 24 months. These vocalizations are preceded and accompanied by increasingly elaborate nonverbal language, from social smiles to descriptive gestures, which serve to augment social connections (Bornstein et al., 2016, Windsor et al., 2009). Self-regulation encompasses a wide range of behaviors in humans, but during early childhood the focus is largely on emotional and behavioral regulation (Putnam et al., 2014). In the first years of life, children co-regulate with familiar caregivers, imitating and responding to both subtle and overt cues (Blair and Raver, 2015). With age, the infant demonstrates increasing autonomy in self-regulation by deliberately communicating needs and desires, moving toward objects of interest when bored, initiating social interactions to seek reassurance, and self-soothing when distressed or hurt. Critically, language and self-regulation skills are not mutually exclusive; rather, these behaviors have transactional relations wherein language promotes development of self-regulation, and vice versa (Chow and Wehby, 2018, Roberts et al., 2018).

Cortical expansion, including dendritic growth, synaptic pruning, and white matter myelination, accompany the cognitive behavioral changes that occur in very early childhood. With respect to language, ventral frontal-temporal connections are involved in semantic processing in infancy, while maturation of the dorsal networks appears to support these processes in later childhood (Brauer et al., 2013). Left temporal lobe specialization for language increases with age, and is simultaneously a mechanism for increased attention to, and encoding of, the native language. Reduced lateralization of the language cortex in children is associated with deficits in both verbal and nonverbal language abilities (Eyler et al., 2012). Myelination in frontal-temporal tracts directly correlates with language production in toddlers (Pujol et al., 2006), although this may simply be an index of age-related growth rather than a causal mechanism for language development (Schlaggar and Aslin, 2006). Emotion regulation, unlike language, or even emotion generation, involves bilateral activations of the frontal and parietal cortices, ventral anterior cingulate, and striatum (Turnbull and Salas, 2021). In infants and young toddlers, the attention orienting neural network is involved in self-regulation, while after three years of age, the executive attention network begins to play a larger role (Posner et al., 2014).

Behaviorally, self-regulation promotes development of language via attention orienting and sustained attention to verbal and nonverbal communication. Moreover, cortical regions activated during deliberate use of emotion regulation strategies by adults overlap with cortical language areas, including the anterior middle cingulate gyrus, supplementary motor area, and bilateral inferior frontal gyrus (Kohn et al., 2014). Among children, greater inhibitory control has been found to be associated with higher receptive vocabulary (Wolfe and Bell, 2004). Likewise, language is an effective means by which children and adults regulate emotions and improve executive functioning, for example, via cognitive reappraisal, self-talk, planning and organizing. Importantly, the association between language abilities and self-regulation in childhood may be moderated by the immediate social environment, for example, emotion socialization by caregivers (Cole et al., 2010). Likewise, the child’s individual temperament and self-regulation skills may mediate the effect of parental emotionality on neurocognitive correlates of self-control (Meza-Cervera et al., 2022).

Electroencephalography (EEG) is a non-invasive method of measuring post-synaptic, extracellular potentials of neuronal populations. EEG metrics can be used as proxies for cortical maturation, thalamocortical network excitation and inhibition, and myelination. Resting EEG power spectral density (PSD) reveals strengths of periodic neural oscillatory activity across a range of frequencies. From infancy through late childhood, the power of slow oscillations decreases and that of faster oscillations increases (Marshall et al., 2002). This is likely due to a number of maturational changes, including synaptic pruning, increased myelination, and greater connectivity both within and between localized neural networks (Betzel et al., 2014). The peak frequency of alpha oscillations increases rapidly from infancy (approximately 5–6 Hz at five months) through adolescence (approximately 10 Hz at 16 years; Freschl et al., 2022a), likely a direct result of myelination and white matter density in thalamocortical feedback circuitries (Segalowitz et al., 2010) (Lőrincz et al., 2009). Moreover, recent work suggests that variability in peak-to-peak timing of alpha oscillations is a marker of healthy brain development (Cole and Voytek, 2019). Alpha power increases at rest, suggesting reduced cognitive engagement of the neural generators (Klimesch, 1999, Fonseca et al., 2013). Successful emotion regulation and executive functioning have been linked to greater left frontal alpha asymmetry, wherein alpha power is stronger in the left frontal cortex relative to the right (Keune et al., 2015, Peltola et al., 2014 Reznik and Allen, 2018).

Periodic oscillatory activity can be differentiated from non-oscillatory, spontaneous cortical signals using programs such as FOOOF (Donoghue et al., 2020). One aperiodic parameter of interest is the 1/f exponent, a non-linear measure of the steepness of the aperiodic spectral slope, i.e., the relative strength of aperiodic activity in slow to faster frequencies. Several investigations have linked atypical aperiodic EEG activity to symptoms of behavioral and social dysregulation (Shuffrey et al., 2022, Wilkinson and Nelson, 2021), as well as states of cognitive arousal (Arnett et al., 2022a). This small body of literature suggests that during early childhood, the 1/f exponent undergoes several developmental changes, before decreasing steadily from early adolescence onward (Hill et al., 2022, Ostlund et al., 2021, McSweeney et al., 2021).

A number of individual differences are known to influence development of language, self-regulation, and cortical maturation. It is well documented that female children develop verbal and nonverbal communication more quickly than male children. However, a recent review suggests that this is due to greater inter-individual variability in language development among males (Rinaldi et al., 2023), rather than reduced language in this group on average. This concept has been replicated with respect to attention abilities in older children (Arnett et al., 2015) and suggests greater individual differences in the rates of cognitive growth among males. Studies of sex differences in neurobiological correlates of language have returned mixed results, but generally support greater left-hemisphere lateralization of receptive language in females compared to males (Etchell et al., 2018). In New Zealand, a large pre-birth cohort study (Growing Up in New Zealand) showed mean differences in toddlers’ expressive vocabulary as a function of sex, socioeconomic status, bilingualism, and temperament. Larger English vocabulary was associated with female sex, first-born status, monolingualism, greater maternal education, more resourced neighborhood, and reduced maternal concerns about the child’s language development. Greater nonverbal communication in infants was associated with greater mother-rated positive affect, surgency, and orienting capacity; and lower negative emotionality (Peterson et al., 2017).

The current study describes neurophysiological (resting EEG) correlates of demographic characteristics, language abilities, and self-regulation in a large, linguistically and socioeconomically diverse sample of infants in New Zealand. We report on cortical functioning in this sample collected at baseline, prior to the start of a blinded clinical trial in which the children are randomly assigned to childcare-based interventions focusing on language development and/or self-regulation. This study is unique with respect to the size of the sample, the inclusion of bilingual infants, the use of modern EEG analytic approaches, and the inclusion of both parent- and teacher-informants.

## 1 Methods

### 1.1 Participants

Participants were recruited from the *Kia Tīmata Pai* The Best Start (KTP) study, which enrolled n = 1481 children aged between 13 and 30 months during the years 2020 - 2022. KTP is a cluster randomized controlled trial conducted through early childhood centers in New Zealand (sampling strategy described elsewhere; Reese et al., 2023). Data were collected only from those children whose parents gave consent. KTP is registered with the Australian New Zealand Clinical Trial Registry (ANZCTR) as ACTRN12621000845831. The Best Start trial and the two sub-studies (Video Project; Brain and Behavior Development [EEG sub-study]) were approved by the University of Otago Health Ethics Committee (H20/116) and reviewed for cultural responsiveness by the Ngāi Tahu Research Committee (University of Otago) and the Māori Advisory Group (University of Auckland, Liggins Institute).

All participants from the main trial were invited to complete the EEG sub-study. A total of 235 children were scheduled to attend the EEG assessment; 42 were not included in the final sample due to not attending the visit (n = 24), attending the visit but not tolerating the EEG net (n = 11), technical failure (n = 1), or insufficient baseline data (n = 6). Thus, the final sample included 193 children aged 15 - 33 months at the baseline EEG assessment. After data processing, all of these participants provided at least 40 s of valid resting EEG (*M* = 121, *SD* = 72, range = 300 - 430 s). Participant characteristics are summarized in Table 1.

**Table 1:** Demographic Characteristics and Data Counts of Participants with and without Resting EEG Data at Baseline.

Variable	Included^a^*N (%)* or *M* (*SD*)	Excluded^b^*N (%)* or *M* (*SD*)	p-value^c^
*N (%)*	193	42	
Sex	192	41	
Female	87 (45%)	18 (44%)	.869
Age	193	42	
Months	20.8 (3.3)	20.5 (3.1)	.674
Ethnicity^d^	190	42	
Māori	36 (19%)	12 (29%)	.164
Pacific Islander	18 (9%)	2 (5%)	.325
Asian	49 (26%)	5 (12%)	.054
Middle Eastern, Latin American or African (MELAA)	4 (2%)	1 (2%)	.911
European	138 (73%)	37 (88%)	.035
Language spoken at home	190	42	
Non-English	30 (16%)	6 (14%)	.808
Biological or adopted child	124	16	
Adopted	2 (2%)	0 (0%)	.609
Born pre-term or on-time	185	40	
Preterm	27 (15%)	5 (13%)	.731
Socioeconomic Status	188	42	.064
Low	16 (9%)	5 (12%)	
Middle	63 (34%)	21 (50%)	
High	109 (58%)	16 (38%)	

Note.

a Included in the final sample as they had valid baseline EEG data.

b Did not have valid baseline data.

c Categorical characteristic variables were compared using chi-squared tests; continuous variables using a t-test.

d We used total count ethnicity, such that each is contrasted with those who do not identify as that ethnicity (e.g., Māori versus non-Māori).

The characteristics of children included and excluded from the final sample were similar, with no significant differences by sex, age, main language spoken at home, adopted status, or premature birth (Table 1). However, compared to the included group, excluded children had a higher proportion of European (vs non-European) children, a nonsignificant trend for a lower proportion of Asian children, and a nonsignificant trend for higher proportions of children from low and middle (vs high) socioeconomic backgrounds.

### 1.2 Procedures

Children enrolled in the EEG sub-study traveled with their caregivers to one of two testing centers in Auckland or Christchurch, New Zealand to complete the EEG assessment. Caregivers provided consent prior to completion of study procedures and were reimbursed for their time and travel expenses. The study visits lasted 75 min and included approximately 30 min of EEG recording, 20 min of parent-child Interaction tasks, and 10 min of cognitive-behavioral testing. The resting EEG paradigm included 3 min (n = 157) or 5 min (n = 36) of baseline recording. During baseline, the child sat on their caregiver’s lap 70 cm from a computer monitor and watched a video of attractive toys. The length of the video and accompanying sound varied across participants, due to changes in the task during a piloting phase. Specifically, the resting EEG paradigm included 5 min of the video with accompanying sound (n = 36), 3 min of video without any sound (n = 16), or 3 min of video with sound during the first 1.5 min and without sound for the remainder (n = 141). Sounds were consistent with the visual display of toys, such as spinning and rolling sounds. The sound volume ranged from 39.2–68.2 dB(A).

Parent and teacher ratings of the child’s language and temperament were collected up to 6 months before or after the EEG acquisition as part of the larger study. Parent ratings were provided by a single caregiver. Teacher ratings were provided by a single teacher, except for 13 children, for whom more than one teacher rating was provided and averaged. To account for the discrepancy between the time of the rating scales and EEG measurement, all language scales were converted to an age-standardized Z score using the child’s age at the time of questionnaire completion. Participants whose language ratings were completed more than 3 months (90 days) before or after the EEG were excluded from these analyses (n = 51 for caregiver reports; n = 29 for teacher reports). A total of 163 participants with EEG data also had valid language data from at least one informant. No adjustment was made to temperament ratings given previously published literature indicating that parent-report of temperament is stable over childhood, particularly for measures of self-regulation (Kopala et al., 2018).

### 1.3 EEG acquisition and processing

Continuous EEG data were acquired using high-density 128 channel geodesic sensor nets with eye electrodes removed, integrated with a 400 series Magstim EGI amplifier and recorded with Netstation 5.4.2 software (Magstim EGI, Eugene, OR). Electrode impedances were reduced to below 50 kOhms prior to recording. EEG signals were referenced to the vertex electrode, analog filtered (0.1 Hz high-pass, 100 Hz elliptical low-pass), amplified and digitized with a sampling rate of 1000 Hz. EEG data were processed and cleaned offline in MATLAB R2020b, using functions from the EEGLAB 2021.1 and ERPLab 8.10 packages. Channels were reduced to the standard 10–20 system (i.e., 20 electrodes including the vertical reference) to optimize the subsequent independent component analysis (ICA) for brief EEG recording. ICA performs best with a high sample:channel ratio (Luck, 2014). Data were downsampled to 250, bandpass filtered at 0.3 and 80 Hz, with 50 Hz line noise and 25 Hz harmonic noise removed using the Cleanline plugin for EEGLAB. Noisy channels were rejected based on amplitudes > 3 standard deviations from the average log power. Recording periods with high artifact (e.g., related to excessive movement) were identified in the continuous data and rejected using an artifact subspace reconstruction algorithm (Arad et al., 2018) with a conservative rejection threshold of 10 standard deviations. The data were referenced to the average and removed channels were then interpolated. Artifactual noise was identified using ICA with a rank order of one fewer than the number of uninterpolated channels. Components with high probability of artifact (*e.g*., eye blinks, line noise, cardiac signal) were removed consistent with previously published pipelines (Levin et al., 2018).

## 2 Measures

### 2.1 Demographics

Demographic information was collected via caregiver-completed questionnaire, either on paper or online. Children were counted as bilingual in the current study if their caregivers indicated that a language other than English was the primary language spoken at home. A three-category socioeconomic status (SES) index (low, middle, high) was derived using the caregiver’s reported occupation, following the New Zealand socio-economic Index (Boven et al., 2022).

### 2.2 Communication skills

Caregivers and teachers completed the New Zealand Communicative Development Inventory (CDI; Peterson et al., 2017; Reese et al., 2018) as a measure of both verbal and nonverbal communication. The CDI includes a checklist of 12 gestures (Fenson et al., 1994) with frequency of gesture use rated on a 3-point Likert scale (0 = Not yet; 1 = Sometimes; 2 = Often). Verbal abilities on the CDI were measured with an expressive vocabulary questionnaire consisting of 100 English words, rated by caregivers and teachers, using a binary scale indicating whether the child speaks each word (0 = Not yet; 1 = Yes). Lastly, the child’s syntax level was measured as their frequency of combining words, assessed through the single question, “Has your/this child started putting words together (in any language), like 'more banana' or 'doggie bite'?” (Fenson et al., 2000). Caregivers and teachers rated this question on a 3-point Likert scale (0 = not yet; 1 = sometimes; 2 = often). Additional details and internal reliability of the communication questionnaires are reported in Supplementary Materials.

### 2.3 Temperament and self-regulation

The parent and teacher versions of the Early Childhood Behaviour Questionnaire Very Short Form (ECBQ; Putnam et al., 2014) are designed to assess child temperament, which includes self-regulation. The questionnaire comprises 36 items deriving scores for three temperament domains: *Negative Affect, Surgency,* and *Effortful Control.* Caregivers rated each question on a 7-point Likert-type scale (ranging from 0 to 6), indicating how often a particular behavior was observed in their child. Scores for each domain were calculated by averaging responses on the 12 relevant items. Additional details are described in Supplementary Materials.

### 2.4 Traditional spectral power

EEG data from 1–80 Hz were fast Fourier transformed using Welch’s method, with a 1-second hamming window and 50% overlap (Jwo et al., 2021). Traditional EEG spectral power was computed as log-transformed power in frequency bands corresponding to theta (4–6 Hz), alpha (6–9 Hz), low beta (12–20 Hz), high beta (20–30 Hz), and gamma (30–45 Hz) frequencies in frontal (Fz,F3, F4), central (Cz, C3, C4), parietal (Pz, P3, P4) and occipital (Oz, O3, O4) regions. These frequency ranges are consistent with the extant literature in this age range (Saby and Marshall, 2012), with the exception of the gamma band, for which the high frequency limit was set at 45 rather than 50 Hz to avoid inclusion of residual line noise.

### 2.5 Adjusted periodic spectral power

Aperiodic spectral power was extracted across 1–45 Hz frequencies using the Fitting Oscillations and One-Over-f (FOOOF) MATLAB toolbox (Donoghue et al., 2020b), following fast Fourier transformation with Welch’s method. FOOOF parameters were specified consistent with prior pediatric EEG literature, (Ostlund et al., 2021, Robertson et al., 2019), including peak width limits of 2–5 Hz, a maximum of 3 peaks, minimum peak height of 1 µV^2^/Hz, and a peak threshold of 2.0 standard deviations beyond the aperiodic data. Adjusted periodic spectral power was then computed as the aperiodic power subtracted from traditional spectral power (log^10^ scale) at each electrode and frequency. When the traditional power value at a particular frequency and electrode was less than the smoothed aperiodic spectrum, the adjusted value was set equal to the original traditional power. This affected 1% of frequencies per individual per electrode. Mean adjusted periodic power was averaged across theta, alpha, low beta, and high beta frequency bands, at midline frontal, central, parietal, and occipital electrode clusters.

### 2.6 Aperiodic exponent

The aperiodic exponent was extracted for each electrode and individual using the FOOOF toolbox and averaged across electrodes at frontal, central, parietal, and occipital regions.

### 2.7 Frontal alpha asymmetry

Frontal alpha asymmetry was calculated as the hemispheric difference in adjusted periodic alpha band power averaged over right frontal (F4 and F8) and left frontal (F3 and F7) electrodes (Brooker et al., 2017, McLaughlin et al., 2011). Higher values indicated more alpha power in the right hemisphere.

### 2.8 Peak alpha frequency

To compute individual peak alpha frequency, EEG data from 1 to 30 Hz were subjected to a Fast Fourier transform with a 1-second hamming window and 50% overlap, in 0.10 Hz increments. Alpha peaks were identified at each of the midline electrodes (Fz, Cz, Pz, Oz) using the pracma library in R Studio 2022.12.0, with peaks defined as the highest log power between 4.0 – 9.0 Hz for which there was at least one decreasing or even point on either side. When multiple peaks met these criteria, the peak with the lowest frequency was selected. 20% of peaks were visually inspected at random to confirm accuracy of the algorithm. For individuals for whom no peaks were identified at any electrode (n = 3), peaks were identified by visual inspection if present. Lastly, an average peak frequency was calculated for each individual as the mean peak frequency across non-missing data from the four midline electrodes.

### 2.9 Analytic plan

Analyses were conducted in R Studio 2022.12.0 using the lme4, lmerTest, and psych packages. Continuous data were first examined for normality. Six outliers in adjusted periodic theta power and four outliers in aperiodic exponent were winsorized to within three standard deviations of the mean. Subsequently, all variables had a normal distribution, with skew and kurtosis values < |3.0|. None of the EEG variables differed by data collection site, nor were they correlated with the amount of resting data available.

The objective of this paper was to describe cross-sectional associations between EEG measures and demographic, reported communication, and temperament variables. We computed a series of linear models with EEG as the dependent variable. Independent variables in the first models were demographics; next, we added language and temperament ratings, while retaining significant demographic covariates, to determine whether language and temperament explained variance in the EEG over and above demographic contributions. Detailed model results are reported in Supplementary Materials. Results pertinent to our research aims are described in the text.

Linear regressions were estimated for demographic analyses with a single EEG outcome measurement (frontal alpha asymmetry and average alpha peak frequency). Multilevel linear models with a random intercept for individual were estimated for repeated measures analyses, *e.g*., across scalp regions and/or frequency bands (traditional spectral power, adjusted periodic power, and aperiodic exponent). Each frequency band has been associated with distinct cognitive and developmental processes in children (Loo et al., 2016); thus, we also included interaction terms between frequency band and age, sex, and SES in our analyses of demographic associations with traditional and adjusted periodic power. A constellation of prior EEG work conducted across multiple developmental stages suggests the aperiodic exponent has a non-linear growth trajectory in early childhood (McSweeney et al., 2021, Schaworonkow and Voytek, 2021, Shuffrey et al., 2022, Hill et al., 2022). Although less well studied, developmental change in the aperiodic exponent may also differ across scalp regions among infants (Schaworonkow and Voytek, 2021). Therefore, in initial analyses of the aperiodic exponent, we tested for both linear and quadratic effects of age, as well as interactions between these growth parameters and scalp region.

In all, four independent models were run for each dependent variable. Due to the exploratory nature of our analyses, and the hypothesis that caregiver and teacher ratings of communication and temperament would show similar associations with EEG metrics, a conservative adjustment was made wherein statistical significance was set as *p* < .0125 (i.e., the traditional alpha = 0.05 was divided by four tests).

### 2.10 Power analyses

Power analyses were conducted in R using functions from the simr library. 1000 simulations were conducted for each effect, using a simulated dataset with categorical and age distributions that mirrored our sample. In our most complex multilevel model (i.e., spectral power as the dependent variable, with fixed main and interaction effects of demographics and three temperament ratings, as well as a random intercept for individual), we had > 97% power to detect a small categorical main effect (*β* = 0.10) and a small categorical-by-continuous interaction (*β* = 0.10), and > 80% power to identify a large continuous main effect (*β =* 0.40), with a significance level of alpha = .0125.

## 3 Results

### 3.1 Traditional spectral power

#### 3.1.1 Demographics

**Fig. 1:**
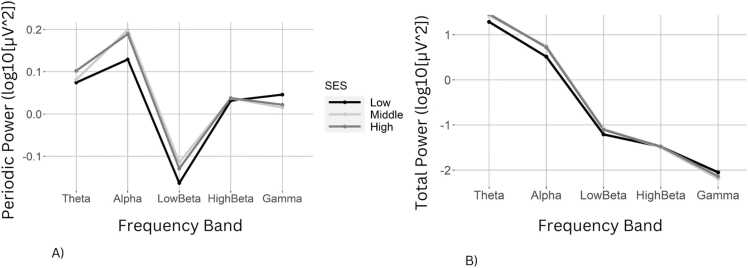
A) Adjusted periodic spectral power by frequency band and socioeconomic status (SES). B) Traditional spectral power by frequency band and SES.

To test for demographic effects on spectral power, we estimated a multilevel linear model with log-transformed power as the dependent variable. Independent variables were frequency band, scalp region, age, sex, and SES; and interactions between frequency band and age, sex, and SES. Reference categories for frequency band and scalp region were alpha and central, respectively. Main effects of power across frequency bands followed the expected trajectory with theta > alpha > low beta > high beta > gamma (*p*s < .001). Relative to the central scalp region, the frontal, parietal, and occipital regions had greater power on average (*p*s < .001). There was an interaction between age and frequency band indicating that power decreased with age in the theta (*p* < .001) band. Males had higher average power than females on average (*p* = .001), but the sex effect was reduced in the gamma frequency band (*p* = .001). There was an interaction between SES and frequency band such that children from low SES families had lower alpha but higher high beta and gamma power band relative to middle and high SES children (*p*s < .002; Fig. 1). Finally, interaction terms indicated bilingualism was associated with reduced power in the gamma relative to alpha band (*p* < .001).

#### 3.1.2 Language

To test for effects of language over and above demographic variables, separate models were run with caregiver- and teacher-ratings of number of gestures, number of English words, and syntax, and their interactions with frequency band, as independent variables. Demographic predictors and interactions were retained in the models. Main effects of caregiver communication ratings were nonsignificant, but interaction effects were apparent. Caregiver-report of greater number of gestures was associated with increased power in the low beta, high beta, and gamma bands relative to alpha (*p*s < .003). Likewise, caregiver ratings of spoken words was associated with increased gamma power (*p* < .001). On the other hand, complexity of syntax as rated by caregivers was associated with reduced gamma and beta powers (*p* < .003). By contrast, there were no associations between teacher-rated communication and traditional EEG power.

#### 3.1.3 Temperament

Caregiver- and teacher-rated temperament domains were added to the model separately to test their associations with spectral power over and above demographic variables. The main effects of caregiver ratings of temperament domains with spectral power were positive. However, interaction effects indicated that caregiver ratings of effortful control and negative affect were more negatively associated with spectral power in the gamma compared to alpha band (*ps* <.007). Main effects of teacher ratings of temperament were not statistically significant, and teacher ratings of effortful control were unrelated to power as main or interaction effects (*p*s > .068). In direct contrast to the results of caregiver ratings, teacher-rated negative affect was positively associated with spectral power in the high beta band (*p* = .006).

### 3.2 Adjusted periodic spectral power

#### 3.2.1 Demographics

**Fig. 2:**
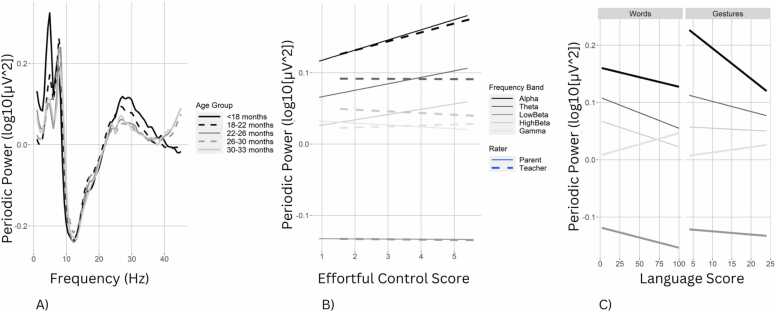
A) Adjusted periodic power spectral density plot by age group. B) Linear associations between adjusted periodic power and effortful control as rated by caregivers (solid line) and teachers (dashed line) by frequency band. C) Linear associations between adjusted periodic power and caregiver-rated vocabulary and gestures.

To test for demographic effects on adjusted periodic power, we repeated the spectral power analyses with periodic power as the dependent variable. Periodic power differed across frequency bands with theta > alpha > low beta (*p*s < .008), but gamma and high beta periodic powers did not statistically differ from alpha. Relative to the central scalp region, periodic power was weaker in the frontal region (*p* < .001). The main effect of age indicated an increase in periodic power with development (*p* = .003); however, this effect was moderated by frequency band, with negative age effects in the theta (*p* < .001) and high beta (*p* < .001) frequency ranges (Fig. 2). Periodic alpha power was higher for middle and high SES compared to low SES children (*p*s < .001). However, there were interactions between frequency band and both sex and SES. Specifically, males had lower periodic power than females in the theta and gamma bands (*p*s < .003). Likewise, the effect of low SES on periodic power was negative in the theta, high beta and gamma bands compared to alpha (*p*s < .008). As with traditional spectral power, bilingual children had greater periodic alpha power (*p* = .012), but lower periodic power in the gamma frequency band (*p* < .001).

#### 3.2.2 Language

Ratings of communication were added separately for caregiver and teacher informants. As with traditional spectral power, periodic power was negatively associated with caregiver-reported words and gestures on average (*ps* <.001), while the association with syntax was positive (*p* < .001). Relative to the alpha band, periodic power in all other bands was more positively associated with caregiver-rated words and gestures (*ps* <.001). Yet, as with the main effect, the opposite associations were found for syntax, with negative associations found between caregiver-reported syntax and, low beta, high beta and gamma periodic power relative to alpha (*ps* <.001). No main or interaction effects were found for teacher-rated communication and periodic power. The associations between age and periodic power across frequency bands were reduced with teacher ratings of communication in the model.

#### 3.2.3 Temperament

Caregiver ratings of effortful control and negative affect were positively associated with periodic power (*p*s < .001). However, interaction terms indicated that the associations of effortful control and negative affect with periodic power were more positive in the alpha band compared to the theta, low beta, and gamma bands (*p*s < .010). Likewise, the association between periodic power and caregiver-rated surgency was negative for the gamma band (*p* = .006). Teacher ratings of effortful control were also positively associated with periodic power on average (*p* = .002), and more positively associated with periodic alpha compared to theta, low beta, high beta, and gamma powers (*p*s < .010). A different pattern emerged for teacher-rated negative affect, which was positively associated with high beta periodic power (*p* < .001). Lastly, the association between teacher-rated surgency and periodic power was more positive in the low beta and high beta compared to alpha bands (*p*s < .003).

### 3.3 Frontal alpha asymmetry

#### 3.3.1 Demographics

Age, sex, SES, and bilingualism associations with frontal alpha asymmetry were tested with a linear regression. Children from middle SES families had lower alpha asymmetry (i.e., reduced right hemisphere alpha power) compared to low SES children, but this did not reach statistical significance (*p* = .030). All other comparisons were likewise nonsignificant.

### 3.4 Language

Frontal alpha asymmetry was not associated with caregiver- or teacher-ratings of verbal or non-verbal communication.

### 3.5 Temperament

Frontal alpha asymmetry was not associated with caregiver- or teacher-ratings of temperament.

### 3.6 Peak alpha frequency

#### 3.6.1 Demographics

**Fig. 3:**
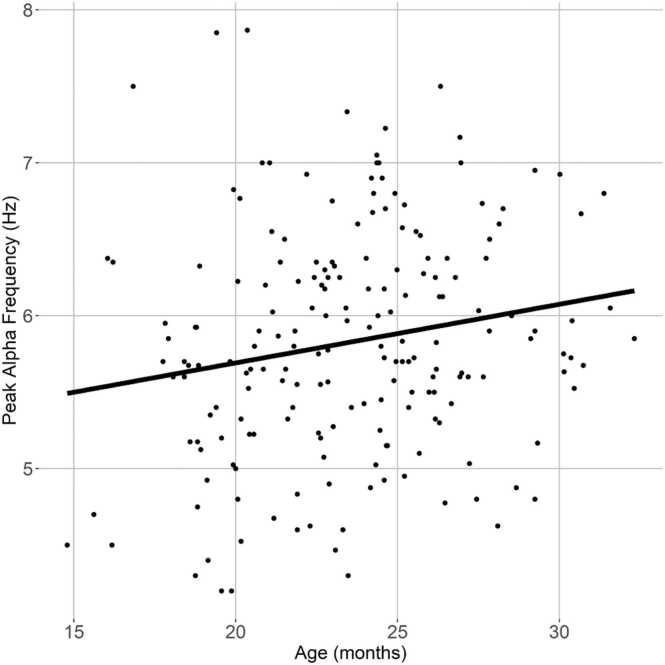
Peak alpha frequency increased with age across participants.

A linear regression was computed with peak alpha frequency (averaged across midline electrodes) as the dependent variable, and age, sex, SES, and bilingualism as independent variables. As expected, age was positively associated with peak alpha frequency; however, this effect did not reach statistical significance (*p* = .036; Fig. 3). None of the other demographic variables showed significant associations.

#### 3.6.2 Language

Neither caregiver- nor teacher-reports of communication were associated with peak alpha frequency over and above demographic variables.

#### 3.6.3 Temperament

No associations between peak alpha frequency and caregiver or teacher ratings of temperament reached statistical significance. With caregiver temperament ratings in the model, the positive association between age and alpha peak frequency was stronger (p = .003).

### 3.7 Aperiodic exponent

#### 3.7.1 Demographics

Main effects of linear and quadratic age were not statistically significant in the initial model; thus, the quadratic age effect was dropped from subsequent analyses. There was a main effect of scalp region wherein the occipital electrode cluster had a higher aperiodic exponent compared to the central region (*p* = .006). Relative to children from low SES backgrounds, children from middle and high SES backgrounds had higher aperiodic exponents on average; although these main effects did not reach statistical significance, they were consistent throughout the remainder of the models. There were no other significant main demographic effects or interactions.

#### 3.7.2 Language

**Fig. 4:**
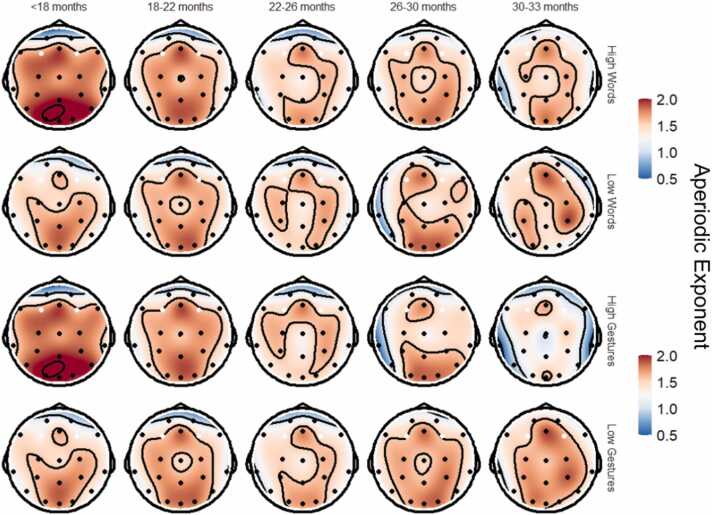
Topographic maps of the aperiodic (1/f) exponent by age group and high versus low caregiver-rated communication scores (using a median split).

Main effects of caregiver-rated words and gestures were not statistically significant (*p*s > .323). However, there was an interaction between caregiver-rated gestures and scalp region wherein the association between aperiodic exponent and gestures was more negative in the occipital than the central scalp (*p* < .001; Fig. 4). No associations between aperiodic exponent and spoken words or syntax were found. Results were similar with teacher-rated words and gestures: in the occipital scalp region, the association between gestures and aperiodic exponent was negative (*p* = .011).

#### 3.7.3 Temperament

Caregiver and teacher ratings of temperament were not associated with aperiodic exponent over and above demographic variables, across all scalp regions.

## 4 Discussion

The KTP study in New Zealand is a large randomized controlled trial in which children are randomly assigned to childcare-based interventions focusing on language development and/or self-regulation. As part of the study, children’s language and self-regulation skills development are being assessed from infancy through early childhood. In the current study, we examined cortical activity in a subset of KTP participants who completed resting EEG at the baseline time point. Our analyses revealed a number of EEG metrics that appear to reflect healthy cortical development in early childhood, as well as indicators specific to development of language and self-regulation.

Cross-sectional analyses of the sample at baseline indicated that adjusted periodic spectral power is more clearly associated with chronological age than is traditional spectral power. In contrast to traditional power calculations, periodic power does not include power contributed by aperiodic neural “noise,” or non-oscillatory, random electrical fluctuations (Donoghue et al., 2020). Therefore, periodic power specifically measures oscillatory burst activity generated by large groups of neurons (Voytek et al., 2015). Unlike the traditional PSD, which generally decreases in power with increasing frequency, the adjusted periodic power distribution highlights a developmentally moderated peak in the beta and gamma ranges (20 – 45 Hz). Periodic beta power in infants has previously been shown to derive in part from transient sensorimotor burst activity which occurs at higher frequencies and shorter durations among adults (Rayson et al., 2022). Likewise, reduced periodic gamma power has been associated with healthy neurodevelopment in early childhood (Wilkinson and Nelson, 2021). High frequency oscillatory changes may reflect cortical pruning in process-specific networks, and increased connectivity of longer, global networks (Schaworonkow and Voytek, 2021). In our age range of 15 - 33 months at the time of EEG acquisition, there was likewise a developmental shift wherein a dominant peak in the “theta” range decreased simultaneously with increased power in the traditional “alpha” range. This appears to reflect maturation of the alpha signal, consistent with previous literature (Freschl et al., 2022). In adults, alpha peak frequency is driven by the speed of information transmission within thalamocortical excitatory-inhibitory feedback loops (Hülsdünker et al., 2016). It is likely that maturation of these circuitries reflects increased myelination and/or synaptic efficiency in thalamocortical connections.

We found that children from low SES families had reduced traditional and adjusted periodic oscillatory power in the alpha frequency band, and greater power in the low beta, and gamma frequencies. The interaction between SES and theta power was only statistically significant for adjusted periodic power. Altogether, these results are consistent with previous studies that have examined associations between traditional EEG spectral power and SES in infants and young children (Maguire and Schneider, 2019, Marshall and Fox, 2004). Unlike previous research, the current study provides novel analyses of SES-associated differences in adjusted periodic as well as traditional spectral power. The consistent results across methods indicate that prior findings are likely driven by oscillatory, rather than aperiodic cortical activity.

Previous research has suggested that in the first few years of life, frontal alpha activity shifts from left to right hemisphere dominance in healthy development (Gabard-Durnam et al., 2015). In our study, middle and higher SES children had greater right-lateralized frontal alpha activity than low SES children, but these differences did not reach statistical significance. We also did not find an association between SES and another reliable measure of cortical maturation, alpha peak frequency, consistent with prior literature (Candelaria-Cook et al., 2022). SES is a broad indicator of many genetic and environmental factors that can influence brain development, such as nutrition, trauma, learning disorders, and exposure to language and reading. As we follow this cohort over time, we anticipate that we will be able to capitalize on the longitudinal study design and randomized intervention to identify both risk and protective factors associated with cortical growth.

One of the primary goals of the KTP parent study is to examine mechanistic effects of childcare-based interventions on language development. Thus, we investigated whether EEG was associated with individual differences in language at baseline. Our study is unique in that we examined rating scales from both caregiver- and teacher-informants, and we included measurement of both verbal and non-verbal communication. Unlike some previous studies (*e.g*., Nordahl-Hansen et al., 2013), inter-rater agreement between caregivers and teachers was low, suggesting that raters were capturing different information about the child’s development. Indeed, results were somewhat inconsistent across caregiver- and teacher-ratings, with EEG metrics generally more strongly associated with caregivers’ reports of communication. Consistent with prior research (Benasich et al., 2008, Cantiani et al., 2019, Wilkinson and Nelson, 2021), periodic gamma band power was positively correlated with verbal and nonverbal language ratings even after accounting for demographic variables. Interestingly, *reduced* periodic alpha power was associated with more caregiver-reported words and gestures. Together, these results indicate that language-related changes in cortical oscillatory power are at least partially distinct from age-related neurodevelopment.

There is limited EEG research on cortical development among bilingual children. Bilingual children in our sample had reduced gamma power and increased alpha power, and these associations remained significant even when communication ratings were included in the model. This finding is consistent with language mapping studies reporting diffuse representations of languages in the brains of bilingual patients (Cervenka et al., 2011). This could mimic the oscillatory patterns of a more immature brain, i.e., reduced localization of functional language networks. Moreover, bilingual children often demonstrate attrition of their home language once they are enrolled in childcare settings (Hammer et al., 2008), which could require cortical reorganization that presents similarly to delayed development.

We examined temperament ratings of effortful control as an indicator of behavior and attention regulation. Effortful control at this early age captures behaviors that promote learning and safety, such as persistence and engagement with toys and social interactions, shifting attention when necessary, and responding to environmental cues. This measure is thus well aligned with effortful control in later childhood, which promotes academic and social learning. Periodic (and to an extent, traditional) alpha power was positively associated with greater effortful control as rated by both caregivers and teachers. In contrast, effortful control and periodic power in the gamma, low beta, and theta bands were negatively associated across raters. In other words, language and effortful control had distinct associations with alpha and gamma power. Although quantitative research and psychological theory would suggest that language facilitates better self-regulation (Montroy et al., 2016, Singer and Bashir, 1999, Vallotton and Ayoub, 2011), alpha and gamma band oscillatory activity do not appear to be shared mechanisms for this association.

Caregiver ratings of negative affect revealed a similar pattern, with high negative affect associated positively with periodic alpha power and negatively with periodic high and low frequency powers. However, it is important to remember that high negative affect suggests emotional dysregulation, while high ratings of effortful control indicate strong attention regulation. Thus, altogether these results suggest that unique cortical circuitries are associated with attention versus emotional control. This is consistent with prior reports indicating that variability in temperament profiles are related to distinct patterns of psychopathology (Karalunas et al., 2014, Rutter and Arnett, 2021). In contrast, teacher ratings revealed common patterns among the three temperament constructs, with a profile of broad dysregulation (low effortful control, high negative affect, and high surgency) associated with immature cortical oscillatory patterns (reduced alpha and increased high frequency powers). Of note, follow-up bivariate Pearson correlations revealed that effortful control was negatively correlated with negative affect and surgency with similar effect sizes across both raters (*r*s range: - 0.06 – - 0.30).

Aperiodic cortical activity has gained attention in recent years with increased availability of statistical packages for easy calculation of the aperiodic exponent (Donoghue et al., 2020). The aperiodic exponent has been proposed as a marker of excitatory/inhibitory balance in the cortex (Donoghue et al., 2020), owing to the differential frequencies of excitatory (faster) and inhibitory (slower) cortical inputs. Excitatory, high frequency signals support segregation of complex cortical information over space and time, while slower inhibitory activity is well-suited for neural information integration (Bruining et al., 2020). Thus, the aperiodic exponent changes dynamically in response to and in anticipation of shifting cognitive and environmental conditions (Arnett et al., 2022a, Arnett et al., 2022b). Although the aperiodic exponent declines with age in adulthood and very early infancy, age-related changes across childhood are not linear, and likely reflect relative growth of local versus global cortical networks (Voytek et al., 2015). In the current study, we found that the aperiodic exponent increased cross-sectionally from 15 - 33 months in the frontal electrodes, i.e., the excitatory/inhibitory balance decreased. Moreover, greater caregiver- and teacher-rated vocabulary was associated with a higher aperiodic exponent in the occipital electrodes. This could suggest age- and social-communication related development of inhibitory frontal and posterior networks with age. However, spatial resolution of the EEG is poor and thus the source of these signals is not fully known.

Baseline data from the KTP EEG study provide new insight into the neurodevelopmental correlates of language, temperament, and self-regulation in early childhood. Strengths of the study include the relatively large sample and inclusion of children from varying socioeconomic and linguistic backgrounds. Limitations include under-representation of children from disadvantaged socioeconomic backgrounds, as well as children with significant developmental delays or disabilities. Note also that the current cross-sectional analyses do not provide any causal information regarding the links between sociodemographic factors, neural signatures, and behavioral indicators of language and self-regulation development. Nonetheless, the current analyses help to establish a baseline from which to compare later development- and intervention-related effects of the KTP trial. Future research in this cohort will consider 1) whether the cross-sectional correlates of language and self-regulation identified here are also important longitudinal predictors of healthy or delayed developmental trajectories, and 2) whether those correlates can be used as early indicators of need for targeted interventions.

## 5 Conclusions

Baseline data obtained from the KTP cohort indicate that EEG, particularly periodic spectral power, is sensitive to individual differences in age, SES, temperament, and language in very young children. Results were only partially consistent across caregiver- and teacher-ratings, suggesting that these informants are capturing distinct aspects of early development. Longitudinal measurement with the KTP trial cohort will reveal the extent to which EEG can be used to measure neurocognitive risk and protective factors for psychosocial development in preschool children.

## CRediT authorship contribution statement

**Poulton Richie:** Conceptualization, Funding acquisition, Investigation, Methodology, Supervision, Writing – review & editing. **Reese Elaine:** Conceptualization, Data curation, Funding acquisition, Methodology, Resources, Supervision, Writing – review & editing. **Bakir-Demir Tugce:** Data curation, Formal analysis, Methodology, Project administration, Writing – review & editing. **Trudgen Anita:** Data curation, Project administration, Writing – review & editing. **Arnett Anne B.:** Conceptualization, Formal analysis, Methodology, Writing – original draft. **Guiney Hayley:** Formal analysis, Methodology, Project administration, Writing – review & editing. **O'Sullivan Justin:** Conceptualization, Funding acquisition, Project administration, Writing – review & editing. **Gluckman Peter:** Conceptualization, Funding acquisition, Supervision, Writing – review & editing. **Schierding William:** Conceptualization, Formal analysis, Resources, Writing – review & editing. **Reid Vincent:** Conceptualization, Supervision, Writing – review & editing.

## Declaration of Competing Interest

The authors declare the following financial interests/personal relationships which may be considered as potential competing interests: Anne B. Arnett reports financial support was provided by The University of Auckland Liggins Institute.
