# Supplementary material for: Resting EEG correlates of neurodevelopment in a socioeconomically and linguistically diverse sample of toddlers: Wave 1 of the Kia Tīmata Pai best start New Zealand study
Source: Dev Cogn Neurosci. 2023 Dec 26;65:101336. doi: 10.1016/j.dcn.2023.101336 (PMC10790011; doi:10.1016/j.dcn.2023.101336)
Supplement: Supplementary file 1 — Supplementary material [file mmc1.docx]

Supplementary Materials

METHODS

**Procedures**

**Recruitment & Enrollment:** Centers (n = 138) across New Zealand (broadly defined as located in Northern, Southern and Midland regions) were recruited into the trial and randomized to one of four conditions: 1) Oral language intervention beginning at approximately age 18 months; 2) Self-regulation intervention beginning at approximately age 3; 3) Combined oral language + self-regulation intervention; or 4) Active control to receive resources on other aspects of child development.

**Measures**

**Demographics:** Reported demographics included the child’s date of birth for exact age calculations, sex, language exposure, and socioeconomic status. With respect to language exposure, caregivers were asked which of the following languages their child could understand: English, Te Reo Māori, Samoan, Tongan, Mandarin Chinese, Mandarin Taiwanese, Cantonese, Hindi, or Other. The SES index is calculated on a scale of 1 to 6, with 6 being the highest socio-economic status. For the current study, ratings were collapsed into three categories: low (rating 1 or 2), medium (3 or 4), and high (5 or 6). When caregiver occupation was unavailable, education level was used instead. This index was computed for both primary and secondary caregivers, and the highest rating was used for analyses.

**CDI Gestures**: Example items on the gestures checklist include, "Signals desire to be picked up by extending the arm upwards" and "Independently waves goodbye when someone leaves". The checklist had good to excellent reliability with a Cronbach's alpha score of 0.79 for caregiver-reports and 0.91 for teacher-reports. The inter-rater agreement (using a single rater two-way mixed effects model of intra-class correlation; ICC) was modest with ICC = 0.31, *p* < .001.

**CDI English Words:** The expressive language questionnaire included words in the categories of sound effects, animals, vehicles, toys, food and drink, clothing, body parts, small household items, furniture and rooms, outdoor items, places to go, people, games and routines, action words, descriptive words, words about time, pronouns, question words, locations, quantifiers, and helping words. Cronbach's alpha reliability was 0.98 for both caregiver- and teacher-reports, indicating a high level of consistency in measuring children's vocabulary development. Inter-rater agreement was moderate: ICC = 0.54, *p* < .001.

**Syntax**: Inter-rater agreement estimated with weighted Cohen’s kappa for ordered categorical variables was moderate: κ = 0.52, *p* < .001.

**ECBQ Temperament**: The questionnaire also offers a "does not apply" option for each item, which is helpful for caregivers and teachers who may not have noticed a specific behavior in the child. Questions marked with "does not apply" do not receive any numerical score. Items contributing to the surgency scale measure behaviors related to impulsivity, high-intensity pleasure, activity level, and social confidence. Cronbach's alpha reliability scores for surgency were 0.64 for caregiver-reports and 0.90 for teacher reports, demonstrating moderate and good internal consistencies, respectively. Caregiver- and teacher-reports showed low inter-rater agreement: ICC = 0.18, *p* = .006. The negative affect scale measures negative emotions such as sadness, fear, anger/frustration, discomfort, and reactivity/soothability. Both caregiver- and teacher-reports demonstrated good internal consistency with Cronbach's alpha scores of .69 and .85, respectively, but low inter-rater agreement (ICC = 0.12, *p* = .051). Finally, the effortful control scale measures inhibitory control, attentional control, low-intensity pleasure, and perceptual sensitivity. This is the domain most closely related to self-regulation in children. The Cronbach's alpha reliability score for this scale was 0.74 for caregiver-reports and 0.82 for teacher-reports, demonstrating good internal consistency. Inter-rater reliability was low, with ICC = 0.14, *p* = .025.
