# Supplementary material for: Resting EEG correlates of neurodevelopment in a socioeconomically and linguistically diverse sample of toddlers: Wave 1 of the Kia Tīmata Pai best start New Zealand study
Source: Dev Cogn Neurosci. 2023 Dec 26;65:101336. doi: 10.1016/j.dcn.2023.101336 (PMC10790011; doi:10.1016/j.dcn.2023.101336)
Supplement: Supplementary file 2 — Supplementary material [file mmc2.pdf]

Contents

Supplementary Table 1a: Traditional PSD and Demographics 2
Supplementary Table 1b: Traditional PSD and Parent-Rated Language 3
Supplementary Table 1c: Traditional PSD and Teacher-Rated Language 4
Supplementary Table 1d: Traditional PSD and Parent-Rated Temperament 5
Supplementary Table 1e: Traditional PSD and Teacher-Rated Temperament 6
Supplementary Table 2a: Adjusted Periodic Power and Demographics 7
Supplementary Table 2b: Adjusted Periodic Power and Parent-Rated Language 8
Supplementary Table 2c: Adjusted Periodic Power and Teacher-Rated Language 9
Supplementary Table 2d: Adjusted Periodic Power and Parent-Rated Temperament 10
Supplementary Table 2e: Adjusted Periodic Power and Teacher-Rated Temperament 11
Supplementary Table 3a: Frontal Alpha Asymmetry and Demographics 12
Supplementary Table 3b: Frontal Alpha Asymmetry and Parent-Rated Language 13
Supplementary Table 3c: Frontal Alpha Asymmetry and Teacher-Rated Language 14
Supplementary Table 3d: Frontal Alpha Asymmetry and Parent-Rated Temperament 15
Supplementary Table 3e: Frontal Alpha Asymmetry and Teacher-Rated Temperament 16
Supplementary Table 4a: Peak Alpha Frequency and Demographics 17
Supplementary Table 4b: Peak Alpha Frequency and Parent-Rated Language 18
Supplementary Table 4c: Peak Alpha Frequency and Teacher-Rated Language 19
Supplementary Table 4d: Peak Alpha Frequency and Parent-Rated Temperament 20
Supplementary Table 4e: Peak Alpha Frequency and Teacher-Rated Temperament 21
Supplementary Table 5a: Aperiodic Exponent and Demographics with Quadratic Age Effect 22
Supplementary Table 5b: Aperiodic Exponent and Demographics with Linear Age Effect Only 23
Supplementary Table 5c: Aperiodic Exponent and Parent-Rated Language 24
Supplementary Table 5d: Aperiodic Exponent and Teacher-Rated Language 25
Supplementary Table 5e: Aperiodic Exponent and Parent-Rated Temperament 26
Supplementary Table 5f: Aperiodic Exponent and Teacher-Rated Temperament 27

Supplementary Table 1a: Traditional PSD and Demographics

Formula: logPower ~ EEG_Age * Band + sex * Band + SES * Band + Multilingual *Band + Region + (1 | ID)

Random effects:

Groups	Name	Variance	Std.Dev.
ID	(Intercept)	0.1330	0.3647
Residual		0.1661	0.4076

Number of obs: 3700, groups: ID, 185

Fixed effects:

	Estimate	Std. Error	df	t value	Pr(>|t|)	
Intercept)	-2.258e-02	2.461e-01	2.734e+02	-0.092	0.926970	
EEG_Age	-1.764e-03	8.756e-03	2.722e+02	-0.201	0.840527	
BandGamma	-2.541e+00	1.695e-01	3.488e+03	-14.994	< 2e-16	***
BandHighBeta	-1.794e+00	1.695e-01	3.488e+03	-10.583	< 2e-16	***
BandLowBeta	-1.665e+00	1.695e-01	3.488e+03	-9.822	< 2e-16	***
BandTheta	1.339e+00	1.695e-01	3.488e+03	7.901	3.68e-15	***
sexMale	2.044e-01	6.230e-02	2.722e+02	3.281	0.001171	**
SESMiddle	3.208e-01	1.210e-01	2.722e+02	2.651	0.008503	**
SESHigh	2.602e-01	1.162e-01	2.722e+02	2.239	0.025970	*
Multilingual	1.859e-01	8.479e-02	2.722e+02	2.193	0.029179	*
RegionFrontal	5.292e-01	1.895e-02	3.488e+03	27.925	< 2e-16	***
RegionOccipital	6.457e-01	1.895e-02	3.488e+03	34.072	< 2e-16	***
RegionParietal	2.667e-01	1.895e-02	3.488e+03	14.073	< 2e-16	***
EEG_Age:BandGamma	-1.189e-03	6.038e-03	3.488e+03	-0.197	0.843887	
EEG_Age:BandHighBeta	-1.292e-02	6.038e-03	3.488e+03	-2.140	0.032404	*
EEG_Age:BandLowBeta	-5.856e-03	6.038e-03	3.488e+03	-0.970	0.332158	
EEG_Age:BandTheta	-2.249e-02	6.038e-03	3.488e+03	-3.724	0.000199	***
BandGamma:sexMale	-1.369e-01	4.296e-02	3.488e+03	-3.187	0.001452	**
BandHighBeta:sexMale	-4.289e-02	4.296e-02	3.488e+03	-0.998	0.318160	
BandLowBeta:sexMale	-2.072e-02	4.296e-02	3.488e+03	-0.482	0.629660	
BandTheta:sexMale	-7.521e-02	4.296e-02	3.488e+03	-1.751	0.080104	.
BandGamma:SESMiddle	-4.781e-01	8.345e-02	3.488e+03	-5.729	1.09e-08	***
BandHighBeta:SESMiddle	-3.002e-01	8.345e-02	3.488e+03	-3.598	0.000326	***
BandLowBeta:SESMiddle	-1.328e-01	8.345e-02	3.488e+03	-1.592	0.111560	
BandTheta:SESMiddle	-1.351e-01	8.345e-02	3.488e+03	-1.619	0.105633	
BandGamma:SESHigh	-3.743e-01	8.015e-02	3.488e+03	-4.669	3.13e-06	***
BandHighBeta:SESHigh	-2.616e-01	8.015e-02	3.488e+03	-3.264	0.001108	**
BandLowBeta:SESHigh	-1.330e-01	8.015e-02	3.488e+03	-1.659	0.097113	.
BandTheta:SESHigh	-7.728e-02	8.015e-02	3.488e+03	-0.964	0.335036	
BandGamma:Multilingual	-1.969e-01	5.847e-02	3.488e+03	-3.367	0.000767	***
BandHighBeta:Multilingual	-9.824e-02	5.847e-02	3.488e+03	-1.680	0.093015	.
BandLowBeta:Multilingual	-6.203e-02	5.847e-02	3.488e+03	-1.061	0.288771	
BandTheta:Multilingual	-2.522e-02	5.847e-02	3.488e+03	-0.431	0.666238	

Signif. codes: 0 ‘***’ 0.001 ‘**’ 0.01 ‘*’ 0.05 ‘.’ 0.1 ‘ ’ 1

Supplementary Table 1b: Traditional PSD and Parent-Rated Language

Formula: logPower ~ EEG_Age * Band + sex * Band + SES * Band + Multilingual * Band + Region + Parent_English_words * Band + Parent_gestures * Band + Parent_syntax * Band + (1 | ID)

Random effects:

Groups	Name	Variance	Std.Dev.
ID	(Intercept)	0.1319	0.3631
Residual		0.1646	0.4057

Number of obs: 2540, groups: ID, 127

Fixed effects:

	Estimate	Std. Error	df	t value	Pr(>|t|)	
(Intercept)	-1.242e-01	3.286e-01	1.801e+02	-0.378	0.705938	
EEG_Age	1.042e-03	1.218e-02	1.795e+02	0.086	0.931909	
BandGamma	-3.104e+00	2.264e-01	2.374e+03	-13.709	< 2e-16	***
BandHighBeta	-2.378e+00	2.264e-01	2.374e+03	-10.505	< 2e-16	***
BandLowBeta	-2.140e+00	2.264e-01	2.374e+03	-9.452	< 2e-16	***
BandTheta	1.264e+00	2.264e-01	2.374e+03	5.581	2.66e-08	***
sexMale	1.767e-01	7.933e-02	1.795e+02	2.228	0.027115	*
SESMiddle	3.957e-01	1.528e-01	1.795e+02	2.590	0.010398	*
SESHigh	2.727e-01	1.432e-01	1.795e+02	1.905	0.058375	.
Multilingual	1.647e-01	9.821e-02	1.795e+02	1.677	0.095365	.
RegionFrontal	5.016e-01	2.277e-02	2.374e+03	22.029	< 2e-16	***
RegionOccipital	6.277e-01	2.277e-02	2.374e+03	27.569	< 2e-16	***
RegionParietal	2.532e-01	2.277e-02	2.374e+03	11.119	< 2e-16	***
Parent_English_words	-2.353e-03	2.332e-03	1.795e+02	-1.009	0.314337	
Parent_gestures	4.274e-03	1.042e-02	1.795e+02	0.410	0.682110	
Parent_syntax	7.227e-02	7.278e-02	1.795e+02	0.993	0.322107	
EEG_Age:BandGamma	4.775e-03	8.402e-03	2.374e+03	0.568	0.569860	
EEG_Age:BandHighBeta	-6.281e-03	8.402e-03	2.374e+03	-0.748	0.454812	
EEG_Age:BandLowBeta	2.329e-04	8.402e-03	2.374e+03	0.028	0.977883	
EEG_Age:BandTheta	-3.732e-02	8.402e-03	2.374e+03	-4.442	9.31e-06	***
BandGamma:sexMale	-8.587e-02	5.471e-02	2.374e+03	-1.570	0.116639	
BandHighBeta:sexMale	-3.004e-02	5.471e-02	2.374e+03	-0.549	0.583028	
BandLowBeta:sexMale	1.814e-02	5.471e-02	2.374e+03	0.332	0.740276	
BandTheta:sexMale	3.683e-03	5.471e-02	2.374e+03	0.067	0.946328	
BandGamma:SESMiddle	-6.102e-01	1.054e-01	2.374e+03	-5.790	7.96e-09	***
BandHighBeta:SESMiddle	-3.747e-01	1.054e-01	2.374e+03	-3.556	0.000384	***
BandLowBeta:SESMiddle	-2.207e-01	1.054e-01	2.374e+03	-2.095	0.036316	*
BandTheta:SESMiddle	-1.477e-01	1.054e-01	2.374e+03	-1.402	0.161137	
BandGamma:SESHigh	-4.036e-01	9.873e-02	2.374e+03	-4.088	4.49e-05	***
BandHighBeta:SESHigh	-2.904e-01	9.873e-02	2.374e+03	-2.941	0.003303	**
BandLowBeta:SESHigh	-1.922e-01	9.873e-02	2.374e+03	-1.947	0.051648	.
BandTheta:SESHigh	7.473e-03	9.873e-02	2.374e+03	0.076	0.939671	
BandGamma:Multilingual	-2.452e-01	6.773e-02	2.374e+03	-3.620	0.000300	***
BandHighBeta:Multilingual	-1.524e-01	6.773e-02	2.374e+03	-2.250	0.024539	*
BandLowBeta:Multilingual	-1.128e-01	6.773e-02	2.374e+03	-1.665	0.096081	.
BandTheta:Multilingual	3.750e-02	6.773e-02	2.374e+03	0.554	0.579838	
BandGamma:Parent_English_words	5.508e-03	1.608e-03	2.374e+03	3.425	0.000626	***
BandHighBeta:Parent_English_words	3.463e-03	1.608e-03	2.374e+03	2.153	0.031407	*
BandLowBeta:Parent_English_words	2.554e-03	1.608e-03	2.374e+03	1.588	0.112444	
BandTheta:Parent_English_words	1.763e-03	1.608e-03	2.374e+03	1.096	0.273092	
BandGamma:Parent_gestures	2.347e-02	7.184e-03	2.374e+03	3.267	0.001101	**
BandHighBeta:Parent_gestures	2.735e-02	7.184e-03	2.374e+03	3.806	0.000145	***
BandLowBeta:Parent_gestures	2.217e-02	7.184e-03	2.374e+03	3.086	0.002051	**
BandTheta:Parent_gestures	1.321e-02	7.184e-03	2.374e+03	1.839	0.066102	.
BandGamma:Parent_syntax	-2.088e-01	5.020e-02	2.374e+03	-4.160	3.29e-05	***
BandHighBeta:Parent_syntax	-1.912e-01	5.020e-02	2.374e+03	-3.809	0.000143	***
BandLowBeta:Parent_syntax	-1.504e-01	5.020e-02	2.374e+03	-2.997	0.002755	**
BandTheta:Parent_syntax	-3.335e-02	5.020e-02	2.374e+03	-0.664	0.506496	

Supplementary Table 1c: Traditional PSD and Teacher-Rated Language

```
Formula: logPower ~ EEG_Age * Band + sex * Band + SES * Band + Multilingual * Band + Region +
Teacher_English_words * Band + Teacher_gestures * Band + Teacher_syntax * Band + (1 | ID)

Random effects:
 Groups   Name        Variance Std.Dev.
 ID       (Intercept) 0.1356   0.3682
 Residual             0.1682   0.4101
Number of obs: 2700, groups:  ID, 135

Fixed effects:
                                  Estimate Std. Error         df t value Pr(>|t|)
(Intercept)                     -1.218e-01  3.132e-01  1.922e+02  -0.389 0.697793
EEG_Age                         -5.213e-03  1.100e-02  1.915e+02  -0.474 0.635966
BandGamma                       -2.578e+00  2.156e-01  2.526e+03 -11.956  < 2e-16 ***
BandHighBeta                    -1.876e+00  2.156e-01  2.526e+03  -8.702  < 2e-16 ***
BandLowBeta                     -1.770e+00  2.156e-01  2.526e+03  -8.207 3.56e-16 ***
BandTheta                        1.263e+00  2.156e-01  2.526e+03   5.856 5.36e-09 ***
sexMale                          2.051e-01  7.689e-02  1.915e+02   2.667 0.008297 **
SESMiddle                        3.792e-01  1.452e-01  1.915e+02   2.612 0.009717 **
SESHigh                          3.353e-01  1.375e-01  1.915e+02   2.438 0.015663 *
Multilingual                     2.303e-01  9.762e-02  1.915e+02   2.359 0.019327 *
RegionFrontal                    4.976e-01  2.233e-02  2.526e+03  22.281  < 2e-16 ***
RegionOccipital                  6.160e-01  2.233e-02  2.526e+03  27.585  < 2e-16 ***
RegionParietal                   2.406e-01  2.233e-02  2.526e+03  10.772  < 2e-16 ***
Teacher_English_words            2.750e-03  2.407e-03  1.915e+02   1.143 0.254632
Teacher_gestures                 8.021e-03  8.275e-03  1.915e+02   0.969 0.333598
Teacher_syntax                  -6.176e-02  7.337e-02  1.915e+02  -0.842 0.400952
EEG_Age:BandGamma                1.566e-02  7.578e-03  2.526e+03   2.067 0.038845 *
EEG_Age:BandHighBeta             1.877e-03  7.578e-03  2.526e+03   0.248 0.804389
EEG_Age:BandLowBeta              6.108e-03  7.578e-03  2.526e+03   0.806 0.420302
EEG_Age:BandTheta               -2.042e-02  7.578e-03  2.526e+03  -2.694 0.007106 **
BandGamma:sexMale               -1.978e-01  5.299e-02  2.526e+03  -3.732 0.000194 ***
BandHighBeta:sexMale            -8.228e-02  5.299e-02  2.526e+03  -1.553 0.120579
BandLowBeta:sexMale             -4.256e-02  5.299e-02  2.526e+03  -0.803 0.421936
BandTheta:sexMale               -7.908e-02  5.299e-02  2.526e+03  -1.492 0.135705
BandGamma:SESMiddle             -5.481e-01  1.001e-01  2.526e+03  -5.477 4.74e-08 ***
BandHighBeta:SESMiddle          -3.837e-01  1.001e-01  2.526e+03  -3.835 0.000129 ***
BandLowBeta:SESMiddle           -1.948e-01  1.001e-01  2.526e+03  -1.946 0.051711 .
BandTheta:SESMiddle             -1.297e-01  1.001e-01  2.526e+03  -1.296 0.195023
BandGamma:SESHigh               -4.331e-01  9.475e-02  2.526e+03  -4.571 5.08e-06 ***
BandHighBeta:SESHigh            -3.169e-01  9.475e-02  2.526e+03  -3.344 0.000838 ***
BandLowBeta:SESHigh             -1.763e-01  9.475e-02  2.526e+03  -1.860 0.062936 .
BandTheta:SESHigh               -2.768e-02  9.475e-02  2.526e+03  -0.292 0.770208
BandGamma:Multilingual          -3.246e-01  6.728e-02  2.526e+03  -4.824 1.49e-06 ***
BandHighBeta:Multilingual       -2.163e-01  6.728e-02  2.526e+03  -3.215 0.001321 **
BandLowBeta:Multilingual        -1.441e-01  6.728e-02  2.526e+03  -2.142 0.032301 *
BandTheta:Multilingual          -3.479e-02  6.728e-02  2.526e+03  -0.517 0.605131
BandGamma:Teacher_English_words -1.019e-03  1.659e-03  2.526e+03  -0.614 0.539070
BandHighBeta:Teacher_English_words  8.505e-04  1.659e-03  2.526e+03   0.513 0.608196
BandLowBeta:Teacher_English_words   2.517e-04  1.659e-03  2.526e+03   0.152 0.879428
BandTheta:Teacher_English_words -1.452e-03  1.659e-03  2.526e+03  -0.875 0.381598
BandGamma:Teacher_gestures      -1.161e-02  5.702e-03  2.526e+03  -2.037 0.041777 *
BandHighBeta:Teacher_gestures   -7.267e-03  5.702e-03  2.526e+03  -1.274 0.202664
BandLowBeta:Teacher_gestures    -3.832e-03  5.702e-03  2.526e+03  -0.672 0.501667
BandTheta:Teacher_gestures       2.809e-03  5.702e-03  2.526e+03   0.493 0.622299
BandGamma:Teacher_syntax        -3.227e-03  5.056e-02  2.526e+03  -0.064 0.949121
BandHighBeta:Teacher_syntax     -4.305e-02  5.056e-02  2.526e+03  -0.852 0.394569
BandLowBeta:Teacher_syntax      -3.298e-02  5.056e-02  2.526e+03  -0.652 0.514258
BandTheta:Teacher_syntax         1.760e-04  5.056e-02  2.526e+03   0.003 0.997223

---
Signif. codes:  0 '***' 0.001 '**' 0.01 '*' 0.05 '.' 0.1 ' ' 1
```

Supplementary Table 1d: Traditional PSD and Parent-Rated Temperament

Formula: logPower ~ EEG_Age * Band + sex * Band + SES * Band + Multilingual * Band + Region + Parent_negative_affect * Band + Parent_effortful_control * Band + Parent_surgency * Band + (1 | ID)

Random effects:

Groups	Name	Variance	Std.Dev.
ID	(Intercept)	0.1334	0.3653
Residual		0.1664	0.4079

Number of obs: 3620, groups: ID, 181

Fixed effects:

	Estimate	Std. Error	df	t value	Pr(>|t|)	
(Intercept)	-8.793e-01	3.626e-01	2.619e+02	-2.425	0.015997	*
EEG_Age	-9.136e-03	9.163e-03	2.614e+02	-0.997	0.319675	
BandGamma	-1.923e+00	2.497e-01	3.400e+03	-7.701	1.76e-14	***
BandHighBeta	-1.478e+00	2.497e-01	3.400e+03	-5.920	3.54e-09	***
BandLowBeta	-1.402e+00	2.497e-01	3.400e+03	-5.613	2.15e-08	***
BandTheta	1.711e+00	2.497e-01	3.400e+03	6.853	8.54e-12	***
sexMale	1.733e-01	6.754e-02	2.614e+02	2.566	0.010854	*
SESMiddle	3.271e-01	1.231e-01	2.614e+02	2.657	0.008367	**
SESHigh	2.995e-01	1.186e-01	2.614e+02	2.524	0.012180	*
Multilingual	1.611e-01	8.555e-02	2.614e+02	1.883	0.060771	.
RegionFrontal	5.266e-01	1.917e-02	3.400e+03	27.469	< 2e-16	***
RegionOccipital	6.401e-01	1.917e-02	3.400e+03	33.389	< 2e-16	***
RegionParietal	2.644e-01	1.917e-02	3.400e+03	13.793	< 2e-16	***
Parent_negative_affect	1.002e-01	4.513e-02	2.614e+02	2.220	0.027302	*
Parent_effortful_control	8.101e-02	4.424e-02	2.614e+02	1.831	0.068257	.
Parent_surgency	1.249e-01	5.167e-02	2.614e+02	2.417	0.016346	*
EEG_Age:BandGamma	5.417e-03	6.313e-03	3.400e+03	0.858	0.390942	
EEG_Age:BandHighBeta	-1.016e-02	6.313e-03	3.400e+03	-1.610	0.107590	
EEG_Age:BandLowBeta	-3.383e-03	6.313e-03	3.400e+03	-0.536	0.592046	
EEG_Age:BandTheta	-1.968e-02	6.313e-03	3.400e+03	-3.117	0.001842	**
BandGamma:sexMale	-1.294e-01	4.653e-02	3.400e+03	-2.780	0.005472	**
BandHighBeta:sexMale	-3.949e-02	4.653e-02	3.400e+03	-0.849	0.396119	
BandLowBeta:sexMale	-2.302e-02	4.653e-02	3.400e+03	-0.495	0.620870	
BandTheta:sexMale	-8.211e-02	4.653e-02	3.400e+03	-1.765	0.077736	.
BandGamma:SESMiddle	-4.488e-01	8.482e-02	3.400e+03	-5.291	1.29e-07	***
BandHighBeta:SESMiddle	-2.922e-01	8.482e-02	3.400e+03	-3.446	0.000577	***
BandLowBeta:SESMiddle	-1.210e-01	8.482e-02	3.400e+03	-1.426	0.153840	
BandTheta:SESMiddle	-1.402e-01	8.482e-02	3.400e+03	-1.653	0.098382	.
BandGamma:SESHigh	-3.870e-01	8.173e-02	3.400e+03	-4.736	2.27e-06	***
BandHighBeta:SESHigh	-2.724e-01	8.173e-02	3.400e+03	-3.333	0.000869	***
BandLowBeta:SESHigh	-1.360e-01	8.173e-02	3.400e+03	-1.664	0.096165	.
BandTheta:SESHigh	-8.994e-02	8.173e-02	3.400e+03	-1.100	0.271240	
BandGamma:Multilingual	-1.858e-01	5.895e-02	3.400e+03	-3.152	0.001636	**
BandHighBeta:Multilingual	-9.134e-02	5.895e-02	3.400e+03	-1.549	0.121363	
BandLowBeta:Multilingual	-5.537e-02	5.895e-02	3.400e+03	-0.939	0.347669	
BandTheta:Multilingual	-3.899e-03	5.895e-02	3.400e+03	-0.066	0.947261	
BandGamma:Parent_negative_affect	-8.536e-02	3.110e-02	3.400e+03	-2.745	0.006085	**
BandHighBeta:Parent_negative_affect	-4.234e-02	3.110e-02	3.400e+03	-1.361	0.173455	
BandLowBeta:Parent_negative_affect	-3.531e-02	3.110e-02	3.400e+03	-1.135	0.256303	
BandTheta:Parent_negative_affect	-7.756e-02	3.110e-02	3.400e+03	-2.494	0.012677	*
BandGamma:Parent_effortful_control	-9.047e-02	3.049e-02	3.400e+03	-2.968	0.003020	**
BandHighBeta:Parent_effortful_control	-3.627e-02	3.049e-02	3.400e+03	-1.190	0.234224	
BandLowBeta:Parent_effortful_control	-4.963e-02	3.049e-02	3.400e+03	-1.628	0.103643	
BandTheta:Parent_effortful_control	-4.316e-02	3.049e-02	3.400e+03	-1.416	0.156898	
BandGamma:Parent_surgency	-6.606e-02	3.560e-02	3.400e+03	-1.856	0.063588	.
BandHighBeta:Parent_surgency	-3.794e-02	3.560e-02	3.400e+03	-1.066	0.286580	
BandLowBeta:Parent_surgency	-1.824e-02	3.560e-02	3.400e+03	-0.512	0.608341	
BandTheta:Parent_surgency	-2.907e-02	3.560e-02	3.400e+03	-0.817	0.414261	

Signif. codes: 0 '***' 0.001 '**' 0.01 '*' 0.05 '.' 0.1 ' ' 1

Supplementary Table 1e: Traditional PSD and Teacher-Rated Temperament

Formula: logPower ~ EEG_Age * Band + sex * Band + SES * Band + Multilingual * Band + Region + Teacher_negative_affect * Band + Teacher_effortful_control * Band + Teacher_surgency * Band + (1 | ID)

Random effects:

Groups	Name	Variance	Std.Dev.
ID	(Intercept)	0.1304	0.3610
Residual		0.1702	0.4125

Number of obs: 3320, groups: ID, 166

Fixed effects:

	Estimate	Std. Error	df	t value	Pr(>|t|)	
(Intercept)	-1.215e-01	3.456e-01	2.432e+02	-0.352	0.725500	
EEG_Age	4.364e-03	9.547e-03	2.426e+02	0.457	0.647996	
BandGamma	-2.183e+00	2.422e-01	3.115e+03	-9.014	< 2e-16	***
BandHighBeta	-1.572e+00	2.422e-01	3.115e+03	-6.489	9.99e-11	***
BandLowBeta	-1.409e+00	2.422e-01	3.115e+03	-5.818	6.56e-09	***
BandTheta	1.494e+00	2.422e-01	3.115e+03	6.169	7.75e-10	***
sexMale	2.002e-01	6.675e-02	2.426e+02	2.999	0.002988	**
SESMiddle	3.239e-01	1.275e-01	2.426e+02	2.540	0.011709	*
SESHigh	2.787e-01	1.217e-01	2.426e+02	2.291	0.022846	*
Multilingual	2.204e-01	8.958e-02	2.426e+02	2.460	0.014594	*
RegionFrontal	5.274e-01	2.025e-02	3.115e+03	26.043	< 2e-16	***
RegionOccipital	6.449e-01	2.025e-02	3.115e+03	31.845	< 2e-16	***
RegionParietal	2.696e-01	2.025e-02	3.115e+03	13.314	< 2e-16	***
Teacher_negative_affect	-1.380e-02	4.280e-02	2.426e+02	-0.322	0.747429	
Teacher_effortful_control	-9.226e-03	4.509e-02	2.426e+02	-0.205	0.838039	
Teacher_surgency	2.631e-03	2.947e-02	2.426e+02	0.089	0.928933	
EEG_Age:BandGamma	-7.715e-03	6.695e-03	3.115e+03	-1.152	0.249297	
EEG_Age:BandHighBeta	-2.378e-02	6.695e-03	3.115e+03	-3.552	0.000389	***
EEG_Age:BandLowBeta	-1.265e-02	6.695e-03	3.115e+03	-1.889	0.058952	.
EEG_Age:BandTheta	-2.547e-02	6.695e-03	3.115e+03	-3.804	0.000145	***
BandGamma:sexMale	-1.390e-01	4.681e-02	3.115e+03	-2.970	0.002998	**
BandHighBeta:sexMale	-6.402e-02	4.681e-02	3.115e+03	-1.368	0.171488	
BandLowBeta:sexMale	-4.356e-02	4.681e-02	3.115e+03	-0.931	0.352093	
BandTheta:sexMale	-9.902e-02	4.681e-02	3.115e+03	-2.115	0.034468	*
BandGamma:SESMiddle	-5.234e-01	8.942e-02	3.115e+03	-5.853	5.32e-09	***
BandHighBeta:SESMiddle	-4.006e-01	8.942e-02	3.115e+03	-4.480	7.72e-06	***
BandLowBeta:SESMiddle	-1.812e-01	8.942e-02	3.115e+03	-2.027	0.042784	*
BandTheta:SESMiddle	-1.144e-01	8.942e-02	3.115e+03	-1.279	0.200873	
BandGamma:SESHigh	-3.875e-01	8.533e-02	3.115e+03	-4.541	5.80e-06	***
BandHighBeta:SESHigh	-2.933e-01	8.533e-02	3.115e+03	-3.437	0.000596	***
BandLowBeta:SESHigh	-1.392e-01	8.533e-02	3.115e+03	-1.632	0.102884	
BandTheta:SESHigh	-3.991e-02	8.533e-02	3.115e+03	-0.468	0.640017	
BandGamma:Multilingual	-2.382e-01	6.282e-02	3.115e+03	-3.791	0.000153	***
BandHighBeta:Multilingual	-1.426e-01	6.282e-02	3.115e+03	-2.270	0.023301	*
BandLowBeta:Multilingual	-8.909e-02	6.282e-02	3.115e+03	-1.418	0.156257	
BandTheta:Multilingual	-4.514e-02	6.282e-02	3.115e+03	-0.719	0.472458	
BandGamma:Teacher_negative_affect	2.126e-02	3.002e-02	3.115e+03	0.708	0.478868	
BandHighBeta:Teacher_negative_affect	8.264e-02	3.002e-02	3.115e+03	2.753	0.005939	**
BandLowBeta:Teacher_negative_affect	2.242e-02	3.002e-02	3.115e+03	0.747	0.455178	
BandTheta:Teacher_negative_affect	2.188e-02	3.002e-02	3.115e+03	0.729	0.466095	
BandGamma:Teacher_effortful_control	-4.779e-02	3.162e-02	3.115e+03	-1.511	0.130796	
BandHighBeta:Teacher_effortful_control	-4.922e-02	3.162e-02	3.115e+03	-1.557	0.119644	
BandLowBeta:Teacher_effortful_control	-5.326e-02	3.162e-02	3.115e+03	-1.685	0.092175	.
BandTheta:Teacher_effortful_control	-5.757e-02	3.162e-02	3.115e+03	-1.821	0.068769	.
BandGamma:Teacher_surgency	-8.585e-03	2.067e-02	3.115e+03	-0.415	0.677875	
BandHighBeta:Teacher_surgency	4.634e-02	2.067e-02	3.115e+03	2.242	0.025007	*
BandLowBeta:Teacher_surgency	3.049e-02	2.067e-02	3.115e+03	1.475	0.140254	
BandTheta:Teacher_surgency	2.582e-02	2.067e-02	3.115e+03	1.249	0.211622	

Signif. codes: 0 ‘***’ 0.001 ‘**’ 0.01 ‘*’ 0.05 ‘.’ 0.1 ‘ ’ 1

Supplementary Table 2a: Adjusted Periodic Power and Demographics

```
Formula: PerPower ~ EEG_Age * Band + sex * Band + SES * Band + Multilingual * Band + Region + (1 | ID)

REML criterion at convergence: -5583.3

Scaled residuals:
    Min      1Q  Median      3Q     Max
-4.8273 -0.5345 -0.0291  0.5105  5.3178

Random effects:
 Groups   Name        Variance Std.Dev.
 ID       (Intercept) 0.001136 0.0337
 Residual             0.011493 0.1072
Number of obs: 3700, groups:  ID, 185

Fixed effects:
                                Estimate Std. Error        df t value Pr(>|t|)
(Intercept)                    5.652e-02  3.737e-02  9.109e+02   1.513 0.130722
EEG_Age                        3.889e-03  1.327e-03  9.005e+02   2.931 0.003461 **
BandGamma                     -7.281e-03  4.459e-02  3.488e+03  -0.163 0.870304
BandHighBeta                   3.659e-02  4.459e-02  3.488e+03   0.820 0.412016
BandLowBeta                   -2.406e-01  4.459e-02  3.488e+03  -5.395 7.32e-08 ***
BandTheta                      1.196e-01  4.459e-02  3.488e+03   2.683 0.007342 **
sexMale                        2.112e-02  9.439e-03  9.005e+02   2.238 0.025490 *
SESMiddle                      9.017e-02  1.834e-02  9.005e+02   4.918 1.04e-06 ***
SESHigh                        7.723e-02  1.761e-02  9.005e+02   4.385 1.29e-05 ***
Multilingual                   3.218e-02  1.285e-02  9.005e+02   2.505 0.012413 *
RegionFrontal                 -1.820e-02  4.986e-03  3.488e+03  -3.651 0.000265 ***
RegionOccipital               -8.156e-03  4.986e-03  3.488e+03  -1.636 0.101984
RegionParietal                 5.902e-03  4.986e-03  3.488e+03   1.184 0.236587
EEG_Age:BandGamma             -2.989e-03  1.589e-03  3.488e+03  -1.882 0.059955 .
EEG_Age:BandHighBeta          -7.451e-03  1.589e-03  3.488e+03  -4.691 2.82e-06 ***
EEG_Age:BandLowBeta           -3.631e-03  1.589e-03  3.488e+03  -2.286 0.022309 *
EEG_Age:BandTheta             -7.434e-03  1.589e-03  3.488e+03  -4.680 2.97e-06 ***
BandGamma:sexMale             -3.957e-02  1.130e-02  3.488e+03  -3.501 0.000470 ***
BandHighBeta:sexMale          -3.832e-03  1.130e-02  3.488e+03  -0.339 0.734623
BandLowBeta:sexMale           -2.449e-04  1.130e-02  3.488e+03  -0.022 0.982711
BandTheta:sexMale             -3.491e-02  1.130e-02  3.488e+03  -3.089 0.002025 **
BandGamma:SESMiddle           -1.340e-01  2.196e-02  3.488e+03  -6.104 1.15e-09 ***
BandHighBeta:SESMiddle        -7.562e-02  2.196e-02  3.488e+03  -3.444 0.000579 ***
BandLowBeta:SESMiddle         -2.529e-02  2.196e-02  3.488e+03  -1.152 0.249506
BandTheta:SESMiddle           -8.582e-02  2.196e-02  3.488e+03  -3.909 9.45e-05 ***
BandGamma:SESHigh             -1.068e-01  2.109e-02  3.488e+03  -5.067 4.25e-07 ***
BandHighBeta:SESHigh          -7.220e-02  2.109e-02  3.488e+03  -3.424 0.000625 ***
BandLowBeta:SESHigh           -3.326e-02  2.109e-02  3.488e+03  -1.577 0.114856
BandTheta:SESHigh             -5.670e-02  2.109e-02  3.488e+03  -2.689 0.007204 **
BandGamma:Multilingual        -5.266e-02  1.538e-02  3.488e+03  -3.424 0.000625 ***
BandHighBeta:Multilingual     -1.823e-02  1.538e-02  3.488e+03  -1.185 0.235957
BandLowBeta:Multilingual      -1.249e-02  1.538e-02  3.488e+03  -0.812 0.416891
BandTheta:Multilingual        -2.455e-02  1.538e-02  3.488e+03  -1.596 0.110532
---
Signif. codes:  0 ‘***’ 0.001 ‘**’ 0.01 ‘*’ 0.05 ‘.’ 0.1 ‘ ’ 1
```

Supplementary Table 2b: Adjusted Periodic Power and Parent-Rated Language

Formula: PerPower ~ EEG_Age * Band + sex * Band + SES * Band + Multilingual * Band + Region + Parent_English_words * Band + Parent_gestures * Band + (1 | ID)

Random effects:

Groups	Name	Variance	Std.Dev.
ID	(Intercept)	0.001227	0.03503
Residual		0.011151	0.10560

Number of obs: 2540, groups: ID, 127

Fixed effects:

	Estimate	Std. Error	df	t value	Pr(>|t|)	
(Intercept)	2.123e-01	5.014e-02	5.596e+02	4.234	2.68e-05	***
EEG_Age	4.996e-03	1.856e-03	5.545e+02	2.692	0.007319	**
BandGamma	-1.662e-01	5.894e-02	2.374e+03	-2.820	0.004841	**
BandHighBeta	-1.539e-01	5.894e-02	2.374e+03	-2.611	0.009092	**
BandLowBeta	-4.093e-01	5.894e-02	2.374e+03	-6.945	4.87e-12	***
BandTheta	3.827e-02	5.894e-02	2.374e+03	0.649	0.516157	
sexMale	-1.325e-02	1.208e-02	5.545e+02	-1.096	0.273356	
SESMiddle	1.060e-01	2.328e-02	5.545e+02	4.555	6.44e-06	***
SESHigh	7.375e-02	2.181e-02	5.545e+02	3.382	0.000771	***
Multilingual	3.967e-02	1.496e-02	5.545e+02	2.652	0.008236	**
RegionFrontal	-1.701e-02	5.927e-03	2.374e+03	-2.870	0.004138	**
RegionOccipital	-8.363e-03	5.927e-03	2.374e+03	-1.411	0.158405	
RegionParietal	8.023e-03	5.927e-03	2.374e+03	1.354	0.176012	
Parent_English_words	-1.219e-03	3.552e-04	5.545e+02	-3.431	0.000647	***
Parent_gestures	-8.346e-03	1.587e-03	5.545e+02	-5.259	2.07e-07	***
Parent_syntax	4.811e-02	1.109e-02	5.545e+02	4.339	1.70e-05	***
EEG_Age:BandGamma	-4.840e-03	2.187e-03	2.374e+03	-2.213	0.027001	*
EEG_Age:BandHighBeta	-7.871e-03	2.187e-03	2.374e+03	-3.598	0.000327	***
EEG_Age:BandLowBeta	-2.941e-03	2.187e-03	2.374e+03	-1.345	0.178860	
EEG_Age:BandTheta	-1.188e-02	2.187e-03	2.374e+03	-5.431	6.17e-08	***
BandGamma:sexMale	5.814e-03	1.424e-02	2.374e+03	0.408	0.683160	
BandHighBeta:sexMale	1.902e-02	1.424e-02	2.374e+03	1.335	0.181858	
BandLowBeta:sexMale	2.685e-02	1.424e-02	2.374e+03	1.885	0.059572	.
BandTheta:sexMale	-4.653e-03	1.424e-02	2.374e+03	-0.327	0.743917	
BandGamma:SESMiddle	-1.547e-01	2.743e-02	2.374e+03	-5.638	1.93e-08	***
BandHighBeta:SESMiddle	-8.068e-02	2.743e-02	2.374e+03	-2.941	0.003305	**
BandLowBeta:SESMiddle	-4.731e-02	2.743e-02	2.374e+03	-1.725	0.084732	.
BandTheta:SESMiddle	-1.037e-01	2.743e-02	2.374e+03	-3.781	0.000160	***
BandGamma:SESHigh	-9.677e-02	2.570e-02	2.374e+03	-3.765	0.000171	***
BandHighBeta:SESHigh	-6.770e-02	2.570e-02	2.374e+03	-2.634	0.008498	**
BandLowBeta:SESHigh	-4.893e-02	2.570e-02	2.374e+03	-1.904	0.057056	.
BandTheta:SESHigh	-3.129e-02	2.570e-02	2.374e+03	-1.217	0.223583	
BandGamma:Multilingual	-5.598e-02	1.763e-02	2.374e+03	-3.175	0.001519	**
BandHighBeta:Multilingual	-2.861e-02	1.763e-02	2.374e+03	-1.622	0.104833	
BandLowBeta:Multilingual	-2.674e-02	1.763e-02	2.374e+03	-1.517	0.129489	
BandTheta:Multilingual	-5.357e-03	1.763e-02	2.374e+03	-0.304	0.761317	
BandGamma:Parent_English_words	1.705e-03	4.187e-04	2.374e+03	4.072	4.81e-05	***
BandHighBeta:Parent_English_words	9.927e-04	4.187e-04	2.374e+03	2.371	0.017811	*
BandLowBeta:Parent_English_words	8.067e-04	4.187e-04	2.374e+03	1.927	0.054125	.
BandTheta:Parent_English_words	1.054e-03	4.187e-04	2.374e+03	2.518	0.011871	*
BandGamma:Parent_gestures	8.127e-03	1.870e-03	2.374e+03	4.345	1.45e-05	***
BandHighBeta:Parent_gestures	1.034e-02	1.870e-03	2.374e+03	5.527	3.61e-08	***
BandLowBeta:Parent_gestures	8.719e-03	1.870e-03	2.374e+03	4.662	3.31e-06	***
BandTheta:Parent_gestures	6.851e-03	1.870e-03	2.374e+03	3.663	0.000255	***
BandGamma:Parent_syntax	-5.392e-02	1.307e-02	2.374e+03	-4.126	3.82e-05	***
BandHighBeta:Parent_syntax	-5.568e-02	1.307e-02	2.374e+03	-4.261	2.12e-05	***
BandLowBeta:Parent_syntax	-4.915e-02	1.307e-02	2.374e+03	-3.761	0.000173	***
BandTheta:Parent_syntax	-2.796e-02	1.307e-02	2.374e+03	-2.140	0.032481	*

Signif. codes: 0 ‘***’ 0.001 ‘**’ 0.01 ‘*’ 0.05 ‘.’ 0.1 ‘ ’ 1

Supplementary Table 2c: Adjusted Periodic Power and Teacher-Rated Language

Formula: PerPower ~ EEG_Age * Band + sex * Band + SES * Band + Multilingual * Band + Region + Teacher_English_words * Band + Teacher_gestures * Band + (1 | ID)

Random effects:

Groups	Name	Variance	Std.Dev.
ID	(Intercept)	0.001239	0.03521
Residual		0.011428	0.10690

Number of obs: 2700, groups: ID, 135

Fixed effects:

	Estimate	Std. Error	df	t value	Pr(>|t|)	
(Intercept)	1.091e-01	4.770e-02	6.034e+02	2.287	0.022536	*
EEG_Age	-7.809e-04	1.672e-03	5.976e+02	-0.467	0.640582	
BandGamma	-5.392e-02	5.618e-02	2.526e+03	-0.960	0.337289	
BandHighBeta	-2.223e-02	5.618e-02	2.526e+03	-0.396	0.692401	
BandLowBeta	-2.996e-01	5.618e-02	2.526e+03	-5.333	1.05e-07	***
BandTheta	8.856e-02	5.618e-02	2.526e+03	1.576	0.115070	
sexMale	2.638e-02	1.169e-02	5.976e+02	2.257	0.024391	*
SESMiddle	9.544e-02	2.207e-02	5.976e+02	4.324	1.80e-05	***
SESHigh	7.489e-02	2.090e-02	5.976e+02	3.583	0.000368	***
Multilingual	6.179e-02	1.484e-02	5.976e+02	4.163	3.60e-05	***
RegionFrontal	-1.781e-02	5.819e-03	2.526e+03	-3.060	0.002234	**
RegionOccipital	-1.169e-02	5.819e-03	2.526e+03	-2.010	0.044576	*
RegionParietal	4.155e-03	5.819e-03	2.526e+03	0.714	0.475339	
Teacher_English_words	4.851e-04	3.659e-04	5.976e+02	1.326	0.185472	
Teacher_gestures	1.694e-03	1.258e-03	5.976e+02	1.347	0.178559	
Teacher_syntax	-1.091e-03	1.115e-02	5.976e+02	-0.098	0.922100	
EEG_Age:BandGamma	2.261e-03	1.975e-03	2.526e+03	1.145	0.252280	
EEG_Age:BandHighBeta	-2.563e-03	1.975e-03	2.526e+03	-1.298	0.194463	
EEG_Age:BandLowBeta	6.544e-04	1.975e-03	2.526e+03	0.331	0.740353	
EEG_Age:BandTheta	-5.053e-03	1.975e-03	2.526e+03	-2.559	0.010559	*
BandGamma:sexMale	-4.861e-02	1.381e-02	2.526e+03	-3.520	0.000439	***
BandHighBeta:sexMale	-8.001e-03	1.381e-02	2.526e+03	-0.580	0.562295	
BandLowBeta:sexMale	-2.077e-03	1.381e-02	2.526e+03	-0.150	0.880417	
BandTheta:sexMale	-3.927e-02	1.381e-02	2.526e+03	-2.844	0.004492	**
BandGamma:SESMiddle	-1.334e-01	2.607e-02	2.526e+03	-5.115	3.38e-07	***
BandHighBeta:SESMiddle	-8.878e-02	2.607e-02	2.526e+03	-3.405	0.000672	***
BandLowBeta:SESMiddle	-3.853e-02	2.607e-02	2.526e+03	-1.478	0.139638	
BandTheta:SESMiddle	-9.374e-02	2.607e-02	2.526e+03	-3.595	0.000330	***
BandGamma:SESHigh	-1.011e-01	2.469e-02	2.526e+03	-4.096	4.34e-05	***
BandHighBeta:SESHigh	-7.291e-02	2.469e-02	2.526e+03	-2.953	0.003177	**
BandLowBeta:SESHigh	-3.828e-02	2.469e-02	2.526e+03	-1.551	0.121138	
BandTheta:SESHigh	-4.850e-02	2.469e-02	2.526e+03	-1.964	0.049582	*
BandGamma:Multilingual	-8.461e-02	1.753e-02	2.526e+03	-4.827	1.47e-06	***
BandHighBeta:Multilingual	-5.202e-02	1.753e-02	2.526e+03	-2.968	0.003030	**
BandLowBeta:Multilingual	-3.779e-02	1.753e-02	2.526e+03	-2.155	0.031225	*
BandTheta:Multilingual	-3.984e-02	1.753e-02	2.526e+03	-2.273	0.023120	*
BandGamma:Teacher_English_words	-9.039e-04	4.323e-04	2.526e+03	-2.091	0.036620	*
BandHighBeta:Teacher_English_words	2.621e-05	4.323e-04	2.526e+03	0.061	0.951651	
BandLowBeta:Teacher_English_words	-9.370e-05	4.323e-04	2.526e+03	-0.217	0.828401	
BandTheta:Teacher_English_words	-7.402e-04	4.323e-04	2.526e+03	-1.712	0.086938	.
BandGamma:Teacher_gestures	-2.650e-03	1.486e-03	2.526e+03	-1.783	0.074643	.
BandHighBeta:Teacher_gestures	-1.375e-03	1.486e-03	2.526e+03	-0.925	0.354864	
BandLowBeta:Teacher_gestures	-6.106e-04	1.486e-03	2.526e+03	-0.411	0.681141	
BandTheta:Teacher_gestures	-5.078e-05	1.486e-03	2.526e+03	-0.034	0.972740	
BandGamma:Teacher_syntax	1.011e-02	1.317e-02	2.526e+03	0.768	0.442837	
BandHighBeta:Teacher_syntax	-1.013e-02	1.317e-02	2.526e+03	-0.769	0.441893	
BandLowBeta:Teacher_syntax	-9.257e-03	1.317e-02	2.526e+03	-0.703	0.482342	
BandTheta:Teacher_syntax	3.381e-03	1.317e-02	2.526e+03	0.257	0.797460	

Signif. codes: 0 ‘***’ 0.001 ‘**’ 0.01 ‘*’ 0.05 ‘.’ 0.1 ‘ ’ 1

Supplementary Table 2d: Adjusted Periodic Power and Parent-Rated Temperament

Formula: PerPower ~ EEG_Age * Band + sex * Band + SES * Band + Multilingual * Band + Region + Parent_negative_affect * Band + Parent_effortful_control * Band + Parent_surgency * Band + (1 | ID)

Random effects:

Groups	Name	Variance	Std.Dev.
ID	(Intercept)	0.001102	0.03319
Residual		0.011412	0.10683

Number of obs: 3620, groups: ID, 181

Fixed effects:

	Estimate	Std. Error	df	t value	Pr(>|t|)	
(Intercept)	-1.275e-01	5.454e-02	8.844e+02	-2.337	0.019670	*
EEG_Age	1.879e-03	1.377e-03	8.797e+02	1.365	0.172697	
BandGamma	2.484e-01	6.542e-02	3.400e+03	3.796	0.000149	***
BandHighBeta	1.639e-01	6.542e-02	3.400e+03	2.506	0.012255	*
BandLowBeta	-1.320e-01	6.542e-02	3.400e+03	-2.017	0.043782	*
BandTheta	3.075e-01	6.542e-02	3.400e+03	4.701	2.69e-06	***
sexMale	2.010e-02	1.015e-02	8.797e+02	1.981	0.047941	*
SESMiddle	8.461e-02	1.850e-02	8.797e+02	4.574	5.47e-06	***
SESHigh	8.149e-02	1.782e-02	8.797e+02	4.572	5.52e-06	***
Multilingual	2.546e-02	1.285e-02	8.797e+02	1.981	0.047923	*
RegionFrontal	-1.800e-02	5.023e-03	3.400e+03	-3.584	0.000343	***
RegionOccipital	-7.267e-03	5.023e-03	3.400e+03	-1.447	0.148061	
RegionParietal	6.496e-03	5.023e-03	3.400e+03	1.293	0.195990	
Parent_negative_affect	3.033e-02	6.781e-03	8.797e+02	4.473	8.73e-06	***
Parent_effortful_control	2.820e-02	6.648e-03	8.797e+02	4.242	2.45e-05	***
Parent_surgency	1.668e-02	7.763e-03	8.797e+02	2.149	0.031912	*
EEG_Age:BandGamma	6.168e-05	1.654e-03	3.400e+03	0.037	0.970253	
EEG_Age:BandHighBeta	-6.117e-03	1.654e-03	3.400e+03	-3.699	0.000220	***
EEG_Age:BandLowBeta	-2.477e-03	1.654e-03	3.400e+03	-1.498	0.134252	
EEG_Age:BandTheta	-6.003e-03	1.654e-03	3.400e+03	-3.629	0.000288	***
BandGamma:sexMale	-3.621e-02	1.219e-02	3.400e+03	-2.970	0.002999	**
BandHighBeta:sexMale	-2.298e-03	1.219e-02	3.400e+03	-0.188	0.850511	
BandLowBeta:sexMale	-1.210e-03	1.219e-02	3.400e+03	-0.099	0.920935	
BandTheta:sexMale	-3.618e-02	1.219e-02	3.400e+03	-2.968	0.003023	**
BandGamma:SESMiddle	-1.249e-01	2.222e-02	3.400e+03	-5.621	2.05e-08	***
BandHighBeta:SESMiddle	-7.485e-02	2.222e-02	3.400e+03	-3.369	0.000764	***
BandLowBeta:SESMiddle	-2.174e-02	2.222e-02	3.400e+03	-0.978	0.327917	
BandTheta:SESMiddle	-8.687e-02	2.222e-02	3.400e+03	-3.910	9.43e-05	***
BandGamma:SESHigh	-1.131e-01	2.141e-02	3.400e+03	-5.280	1.37e-07	***
BandHighBeta:SESHigh	-7.736e-02	2.141e-02	3.400e+03	-3.613	0.000307	***
BandLowBeta:SESHigh	-3.485e-02	2.141e-02	3.400e+03	-1.628	0.103664	
BandTheta:SESHigh	-6.337e-02	2.141e-02	3.400e+03	-2.960	0.003101	**
BandGamma:Multilingual	-4.387e-02	1.544e-02	3.400e+03	-2.841	0.004523	**
BandHighBeta:Multilingual	-1.228e-02	1.544e-02	3.400e+03	-0.795	0.426561	
BandLowBeta:Multilingual	-7.844e-03	1.544e-02	3.400e+03	-0.508	0.611520	
BandTheta:Multilingual	-1.604e-02	1.544e-02	3.400e+03	-1.039	0.299049	
BandGamma:Parent_negative_affect	-4.281e-02	8.147e-03	3.400e+03	-5.255	1.57e-07	***
BandHighBeta:Parent_negative_affect	-2.266e-02	8.147e-03	3.400e+03	-2.781	0.005444	**
BandLowBeta:Parent_negative_affect	-1.786e-02	8.147e-03	3.400e+03	-2.192	0.028424	*
BandTheta:Parent_negative_affect	-3.379e-02	8.147e-03	3.400e+03	-4.148	3.44e-05	***
BandGamma:Parent_effortful_control	-3.760e-02	7.986e-03	3.400e+03	-4.708	2.60e-06	***
BandHighBeta:Parent_effortful_control	-1.449e-02	7.986e-03	3.400e+03	-1.815	0.069652	.
BandLowBeta:Parent_effortful_control	-2.081e-02	7.986e-03	3.400e+03	-2.605	0.009217	**
BandTheta:Parent_effortful_control	-2.090e-02	7.986e-03	3.400e+03	-2.617	0.008911	**
BandGamma:Parent_surgency	-2.574e-02	9.326e-03	3.400e+03	-2.760	0.005815	**
BandHighBeta:Parent_surgency	-1.428e-02	9.326e-03	3.400e+03	-1.531	0.125745	
BandLowBeta:Parent_surgency	-6.625e-03	9.326e-03	3.400e+03	-0.710	0.477545	
BandTheta:Parent_surgency	-1.795e-02	9.326e-03	3.400e+03	-1.924	0.054377	.

Signif. codes: 0 ‘***’ 0.001 ‘**’ 0.01 ‘*’ 0.05 ‘.’ 0.1 ‘ ’ 1

Supplementary Table 2e: Adjusted Periodic Power and Teacher-Rated Temperament

Formula: PerPower ~ EEG_Age * Band + sex * Band + SES * Band + Multilingual * Band + Region + Teacher_negative_affect * Band + Teacher_effortful_control * Band + Teacher_surgency * Band + (1 | ID)

Random effects:

Groups	Name	Variance	Std.Dev.
ID	(Intercept)	0.001202	0.03466
Residual		0.011595	0.10768

Number of obs: 3320, groups: ID, 166

Fixed effects:

	Estimate	Std. Error	df	t value	Pr(>|t|)	
(Intercept)	-2.224e-02	5.326e-02	7.717e+02	-0.418	0.676408	
EEG_Age	5.930e-03	1.470e-03	7.668e+02	4.035	6.02e-05	***
BandGamma	8.760e-02	6.323e-02	3.115e+03	1.385	0.166008	
BandHighBeta	8.786e-02	6.323e-02	3.115e+03	1.390	0.164758	
BandLowBeta	-1.562e-01	6.323e-02	3.115e+03	-2.470	0.013547	*
BandTheta	2.329e-01	6.323e-02	3.115e+03	3.684	0.000234	***
sexMale	2.860e-02	1.028e-02	7.668e+02	2.784	0.005508	**
SESMiddle	9.932e-02	1.963e-02	7.668e+02	5.060	5.26e-07	***
SESHigh	7.059e-02	1.873e-02	7.668e+02	3.769	0.000177	***
Multilingual	4.459e-02	1.379e-02	7.668e+02	3.233	0.001277	**
RegionFrontal	-1.845e-02	5.287e-03	3.115e+03	-3.490	0.000490	***
RegionOccipital	-7.730e-03	5.287e-03	3.115e+03	-1.462	0.143811	
RegionParietal	5.498e-03	5.287e-03	3.115e+03	1.040	0.298480	
Teacher_negative_affect	-9.888e-03	6.590e-03	7.668e+02	-1.501	0.133887	
Teacher_effortful_control	2.156e-02	6.941e-03	7.668e+02	3.106	0.001969	**
Teacher_surgency	-1.170e-02	4.537e-03	7.668e+02	-2.579	0.010106	*
EEG_Age:BandGamma	-4.338e-03	1.748e-03	3.115e+03	-2.482	0.013114	*
EEG_Age:BandHighBeta	-1.106e-02	1.748e-03	3.115e+03	-6.329	2.82e-10	***
EEG_Age:BandLowBeta	-5.928e-03	1.748e-03	3.115e+03	-3.392	0.000703	***
EEG_Age:BandTheta	-9.156e-03	1.748e-03	3.115e+03	-5.238	1.73e-07	***
BandGamma:sexMale	-4.381e-02	1.222e-02	3.115e+03	-3.585	0.000342	***
BandHighBeta:sexMale	-1.548e-02	1.222e-02	3.115e+03	-1.267	0.205362	
BandLowBeta:sexMale	-1.163e-02	1.222e-02	3.115e+03	-0.952	0.341392	
BandTheta:sexMale	-4.346e-02	1.222e-02	3.115e+03	-3.556	0.000382	***
BandGamma:SESMiddle	-1.329e-01	2.334e-02	3.115e+03	-5.692	1.38e-08	***
BandHighBeta:SESMiddle	-1.037e-01	2.334e-02	3.115e+03	-4.444	9.14e-06	***
BandLowBeta:SESMiddle	-3.714e-02	2.334e-02	3.115e+03	-1.591	0.111694	
BandTheta:SESMiddle	-8.294e-02	2.334e-02	3.115e+03	-3.553	0.000386	***
BandGamma:SESHigh	-9.566e-02	2.228e-02	3.115e+03	-4.294	1.81e-05	***
BandHighBeta:SESHigh	-7.334e-02	2.228e-02	3.115e+03	-3.292	0.001005	**
BandLowBeta:SESHigh	-2.850e-02	2.228e-02	3.115e+03	-1.279	0.200917	
BandTheta:SESHigh	-4.509e-02	2.228e-02	3.115e+03	-2.024	0.043029	*
BandGamma:Multilingual	-6.518e-02	1.640e-02	3.115e+03	-3.974	7.23e-05	***
BandHighBeta:Multilingual	-3.347e-02	1.640e-02	3.115e+03	-2.041	0.041383	*
BandLowBeta:Multilingual	-2.186e-02	1.640e-02	3.115e+03	-1.333	0.182720	
BandTheta:Multilingual	-3.644e-02	1.640e-02	3.115e+03	-2.222	0.026376	*
BandGamma:Teacher_negative_affect	1.609e-03	7.836e-03	3.115e+03	0.205	0.837314	
BandHighBeta:Teacher_negative_affect	3.022e-02	7.836e-03	3.115e+03	3.856	0.000117	***
BandLowBeta:Teacher_negative_affect	6.382e-03	7.836e-03	3.115e+03	0.814	0.415439	
BandTheta:Teacher_negative_affect	1.050e-02	7.836e-03	3.115e+03	1.340	0.180355	
BandGamma:Teacher_effortful_control	-2.155e-02	8.255e-03	3.115e+03	-2.610	0.009093	**
BandHighBeta:Teacher_effortful_control	-2.197e-02	8.255e-03	3.115e+03	-2.661	0.007830	**
BandLowBeta:Teacher_effortful_control	-2.348e-02	8.255e-03	3.115e+03	-2.845	0.004476	**
BandTheta:Teacher_effortful_control	-2.897e-02	8.255e-03	3.115e+03	-3.509	0.000456	***
BandGamma:Teacher_surgency	3.779e-03	5.395e-03	3.115e+03	0.700	0.483668	
BandHighBeta:Teacher_surgency	2.571e-02	5.395e-03	3.115e+03	4.766	1.97e-06	***
BandLowBeta:Teacher_surgency	1.654e-02	5.395e-03	3.115e+03	3.066	0.002185	**
BandTheta:Teacher_surgency	5.533e-03	5.395e-03	3.115e+03	1.026	0.305196	

Signif. codes: 0 ‘***’ 0.001 ‘**’ 0.01 ‘*’ 0.05 ‘.’ 0.1 ‘ ’ 1

Supplementary Table 3a: Frontal Alpha Asymmetry and Demographics

```
Formula: Frontal_Alpha_Asymm ~ EEG_Age + sex + SES + Multilingual, data = Asymdf)

Coefficients:
                     Estimate Std. Error t value Pr(>|t|)
(Intercept)          0.0534739  0.0466958   1.145   0.2537
EEG_Age             -0.0003599  0.0016634  -0.216   0.8289
sexMale             -0.0108242  0.0118357  -0.915   0.3617
SESMiddle           -0.0502265  0.0229909  -2.185   0.0302 *
SESHigh             -0.0372566  0.0220806  -1.687   0.0933 .
Multilingual         0.0194955  0.0161076   1.210   0.2277
---
Signif. codes:  0 '***' 0.001 '**' 0.01 '*' 0.05 '.' 0.1 ' ' 1

Residual standard error: 0.07939 on 179 degrees of freedom
  (8 observations deleted due to missingness)
Multiple R-squared:  0.03716,     Adjusted R-squared:  0.01027
F-statistic: 1.382 on 5 and 179 DF,  p-value: 0.2332
```

Supplementary Table 3b: Frontal Alpha Asymmetry and Parent-Rated Language

Formula: Frontal_Alpha_Asymm ~ EEG_Age + sex + SES + Multilingual + Parent_English_words + Parent_gestures + Parent_syntax

Coefficients:

	Estimate	Std. Error	t value	Pr(>|t|)
(Intercept)	0.0182623	0.0665103	0.275	0.784
EEG_Age	-0.0012323	0.0024683	-0.499	0.619
sexMale	-0.0074013	0.0160725	-0.460	0.646
SESMiddle	-0.0463607	0.0309591	-1.497	0.137
SESHigh	-0.0416616	0.0290047	-1.436	0.154
Multilingual	0.0120086	0.0198994	0.603	0.547
Parent_English_words	0.0003891	0.0004724	0.824	0.412
Parent_gestures	0.0029624	0.0021106	1.404	0.163
Parent_syntax	-0.0195097	0.0147472	-1.323	0.188

Residual standard error: 0.08427 on 118 degrees of freedom
(66 observations deleted due to missingness)
Multiple R-squared: 0.05633, Adjusted R-squared: -0.007645
F-statistic: 0.8805 on 8 and 118 DF, p-value: 0.5352

Supplementary Table 3c: Frontal Alpha Asymmetry and Teacher-Rated Language

```
Formula: Frontal_Alpha_Asymm ~ EEG_Age + sex + SES + Multilingual + Teacher_English_words +
Teacher_gestures + Teacher_syntax

Coefficients:
                        Estimate Std. Error t value Pr(>|t|)
(Intercept)            0.0328564  0.0603853   0.544   0.5873
EEG_Age                0.0007759  0.0021224   0.366   0.7153
sexMale               -0.0014882  0.0148406  -0.100   0.9203
SESMiddle             -0.0601040  0.0280249  -2.145   0.0339 *
SESHigh               -0.0460631  0.0265385  -1.736   0.0851 .
Multilingual           0.0077225  0.0188427   0.410   0.6826
Teacher_English_words -0.0001172  0.0004646  -0.252   0.8012
Teacher_gestures      -0.0001001  0.0015971  -0.063   0.9501
Teacher_syntax         0.0110449  0.0141608   0.780   0.4369
---
Signif. codes:  0 '***' 0.001 '**' 0.01 '*' 0.05 '.' 0.1 ' ' 1

Residual standard error: 0.08126 on 126 degrees of freedom
  (58 observations deleted due to missingness)
Multiple R-squared:  0.04197,     Adjusted R-squared:  -0.01886
F-statistic:  0.69 on 8 and 126 DF,  p-value: 0.6997
```

Supplementary Table 3d: Frontal Alpha Asymmetry and Parent-Rated Temperament

```
Formula: Frontal_Alpha_Asymm ~ EEG_Age + sex + SES + Multilingual + Parent_negative_affect +
Parent_effortful_control + Parent_surgency

Coefficients:
                          Estimate Std. Error t value Pr(>|t|)
(Intercept)               0.0758022  0.0689580   1.099   0.2732
EEG_Age                  -0.0009872  0.0017434  -0.566   0.5720
sexMale                  -0.0104468  0.0128503  -0.813   0.4174
SESMiddle                -0.0550555  0.0234220  -2.351   0.0199 *
SESHigh                  -0.0411546  0.0225691  -1.823   0.0700 .
Multilingual              0.0180251  0.0162777   1.107   0.2697
Parent_negative_affect    0.0134677  0.0085872   1.568   0.1186
Parent_effortful_control -0.0009129  0.0084181  -0.108   0.9138
Parent_surgency          -0.0066180  0.0098305  -0.673   0.5017
---
Signif. codes:  0 '***' 0.001 '**' 0.01 '*' 0.05 '.' 0.1 ' ' 1

Residual standard error: 0.07677 on 154 degrees of freedom
  (30 observations deleted due to missingness)
Multiple R-squared:  0.05684,     Adjusted R-squared:  0.007848
F-statistic:  1.16 on 8 and 154 DF,  p-value: 0.3268
```

Supplementary Table 3e: Frontal Alpha Asymmetry and Teacher-Rated Temperament

```
Formula: Frontal_Alpha_Asymm ~ EEG_Age + sex + SES + Multilingual + Teacher_negative_affect +
Teacher_effortful_control + Teacher_surgency, data = Asymdf)

Coefficients:
                            Estimate Std. Error t value Pr(>|t|)
(Intercept)                0.0039607  0.0644330   0.061    0.951
EEG_Age                    0.0005653  0.0017812   0.317    0.751
sexMale                   -0.0005681  0.0124533  -0.046    0.964
SESMiddle                 -0.0341258  0.0237886  -1.435    0.153
SESHigh                   -0.0262813  0.0227017  -1.158    0.249
Multilingual               0.0114020  0.0167134   0.682    0.496
Teacher_negative_affect    0.0052716  0.0079860   0.660    0.510
Teacher_effortful_control -0.0028855  0.0084122  -0.343    0.732
Teacher_surgency           0.0045766  0.0054981   0.832    0.406

Residual standard error: 0.07762 on 157 degrees of freedom
  (27 observations deleted due to missingness)
Multiple R-squared:  0.02466,     Adjusted R-squared:  -0.02504
F-statistic: 0.4962 on 8 and 157 DF,  p-value: 0.8576
```

Supplementary Table 4a: Peak Alpha Frequency and Demographics

```
Formula: Frequency ~ EEG_Age + sex + SES + Multilingual, data = Alphadf)

Coefficients:
                   Estimate Std. Error t value Pr(>|t|)
(Intercept)         5.13261    0.43049  11.923   <2e-16 ***
EEG_Age             0.03233    0.01533   2.109   0.0364 *
sexMale            -0.06006    0.10925  -0.550   0.5832
SESMiddle           0.01268    0.21182   0.060   0.9523
SESHigh            -0.03520    0.20360  -0.173   0.8629
Multilingual       -0.08660    0.14848  -0.583   0.5605
---
Signif. codes:  0 '***' 0.001 '**' 0.01 '*' 0.05 '.' 0.1 ' ' 1

Residual standard error: 0.7312 on 178 degrees of freedom
  (8 observations deleted due to missingness)
Multiple R-squared:  0.0326,      Adjusted R-squared:  0.005426
F-statistic:   1.2 on 5 and 178 DF,  p-value: 0.3112
```

Supplementary Table 4b: Peak Alpha Frequency and Parent-Rated Language

```
Formula: Frequency ~ EEG_Age + sex + SES + Multilingual + Parent_English_words +
Parent_gestures + Parent_syntax

Coefficients:
                      Estimate Std. Error t value Pr(>|t|)    
(Intercept)           4.364957   0.553746   7.883 1.85e-12 ***
EEG_Age               0.035624   0.020527   1.735   0.0853 .  
sexMale               0.116960   0.134200   0.872   0.3852    
SESMiddle             0.164187   0.257442   0.638   0.5249    
SESHigh               0.035739   0.241365   0.148   0.8825    
Multilingual         -0.141055   0.165583  -0.852   0.3960    
Parent_English_words  0.002282   0.003929   0.581   0.5626    
Parent_gestures       0.025931   0.017589   1.474   0.1431    
Parent_syntax        -0.137757   0.123016  -1.120   0.2651    
---
Signif. codes:  0 '***' 0.001 '**' 0.01 '*' 0.05 '.' 0.1 ' ' 1

Residual standard error: 0.7006 on 117 degrees of freedom
  (66 observations deleted due to missingness)
Multiple R-squared:  0.07821,     Adjusted R-squared:  0.01519 
F-statistic: 1.241 on 8 and 117 DF,  p-value: 0.2816
```

Supplementary Table 4c: Peak Alpha Frequency and Teacher-Rated Language

```
Formula: Frequency ~ EEG_Age + sex + SES + Multilingual + Teacher_English_words +
Teacher_gestures + Teacher_syntax

Coefficients:
                       Estimate Std. Error t value Pr(>|t|)
(Intercept)            5.066618   0.531636   9.530   <2e-16 ***
EEG_Age                0.035426   0.018686   1.896   0.0603 .
sexMale               -0.040689   0.131292  -0.310   0.7571
SESMiddle              0.176627   0.246739   0.716   0.4754
SESHigh                0.150038   0.233680   0.642   0.5220
Multilingual          -0.141001   0.165885  -0.850   0.3970
Teacher_English_words  0.001427   0.004094   0.348   0.7281
Teacher_gestures      -0.013729   0.014063  -0.976   0.3308
Teacher_syntax        -0.001356   0.125420  -0.011   0.9914
---
Signif. codes:  0 ‘***’ 0.001 ‘**’ 0.01 ‘*’ 0.05 ‘.’ 0.1 ‘ ’ 1

Residual standard error: 0.7152 on 125 degrees of freedom
  (58 observations deleted due to missingness)
Multiple R-squared:  0.04609,     Adjusted R-squared:  -0.01496
F-statistic: 0.755 on 8 and 125 DF,  p-value: 0.6429
```

Supplementary Table 4d: Peak Alpha Frequency and Parent-Rated Temperament

```
Formula: Frequency ~ EEG_Age + sex + SES + Multilingual + Parent_negative_affect + 
Parent_effortful_control + Teacher_surgency

Coefficients:
                          Estimate Std. Error t value Pr(>|t|)    
(Intercept)               6.114809   0.614981   9.943  < 2e-16 ***
EEG_Age                   0.046097   0.015551   2.964  0.00347 ** 
sexMale                   0.016635   0.114885   0.145  0.88505    
SESMiddle                 0.009616   0.208760   0.046  0.96332    
SESHigh                  -0.119509   0.201277  -0.594  0.55346    
Multilingual             -0.047326   0.145145  -0.326  0.74478    
Parent_negative_affect   -0.046945   0.076547  -0.613  0.54050    
Parent_effortful_control -0.100539   0.075171  -1.337  0.18285    
Parent_surgency          -0.200334   0.087660  -2.285  0.02352 *  
---
Signif. codes:  0 ‘***’ 0.001 ‘**’ 0.01 ‘*’ 0.05 ‘.’ 0.1 ‘ ’ 1

Residual standard error: 0.7291 on 153 degrees of freedom
  (30 observations deleted due to missingness)
Multiple R-squared:  0.05201,	Adjusted R-squared:  0.002445 
F-statistic: 1.049 on 8 and 153 DF,  p-value: 0.4018
```

Supplementary Table 4e: Peak Alpha Frequency and Teacher-Rated Temperament

```
Formula: Frequency ~ EEG_Age + sex + SES + Multilingual + Teacher_negative_affect + Teacher_effortful_control + 
Teacher_surgency

Coefficients:
                           Estimate Std. Error t value Pr(>|t|)    
(Intercept)                5.13266    0.62141   8.260 5.86e-14 ***
EEG_Age                    0.02283    0.01716   1.330    0.185    
sexMale                   -0.02831    0.12012  -0.236    0.814    
SESMiddle                 -0.08736    0.22903  -0.381    0.703    
SESHigh                   -0.11286    0.21878  -0.516    0.607    
Multilingual              -0.08516    0.16100  -0.529    0.598    
Teacher_negative_affect    0.06576    0.07690   0.855    0.394    
Teacher_effortful_control  0.02834    0.08100   0.350    0.727    
Teacher_surgency           0.03036    0.05303   0.573    0.568    
---
Signif. codes:  0 '***' 0.001 '**' 0.01 '*' 0.05 '.' 0.1 ' ' 1

Residual standard error: 0.747 on 156 degrees of freedom
  (27 observations deleted due to missingness)
Multiple R-squared:  0.02775,     Adjusted R-squared:  -0.0221 
F-statistic: 0.5567 on 8 and 156 DF,  p-value: 0.812
```

Supplementary Table 5a: Aperiodic Exponent and Demographics with Quadratic Age Effect

```
Formula: Exponent ~ EEG_Age * Region + Age^2 * Region + sex + SES + Multilingual + (1 | ID)

Random effects:
 Groups   Name        Variance Std.Dev.
 ID       (Intercept) 0.03455  0.1859
 Residual             0.06296  0.2509
Number of obs: 2220, groups:  ID, 185

Fixed effects:
                          Estimate Std. Error         df t value Pr(>|t|)
(Intercept)              2.333e+00  6.063e-01  3.406e+02   3.848 0.000142 ***
EEG_Age                 -7.450e-02  5.215e-02  3.356e+02  -1.429 0.154042
RegionFrontal           -1.051e+00  5.257e-01  2.026e+03  -1.999 0.045768 *
RegionOccipital          3.652e-01  5.257e-01  2.026e+03   0.695 0.487371
RegionParietal           6.678e-01  5.257e-01  2.026e+03   1.270 0.204157
Age^2                    1.533e-03  1.097e-03  3.351e+02   1.398 0.163052
sexMale                  3.087e-02  2.975e-02  1.780e+02   1.038 0.300866
SESMiddle                1.224e-01  5.880e-02  1.780e+02   2.082 0.038749 *
SESHigh                  9.468e-02  5.635e-02  1.780e+02   1.680 0.094657 .
Multilingual             5.116e-02  4.049e-02  1.780e+02   1.264 0.208014
EEG_Age:RegionFrontal    9.072e-02  4.477e-02  2.026e+03   2.026 0.042849 *
EEG_Age:RegionOccipital -1.174e-02  4.477e-02  2.026e+03  -0.262 0.793146
EEG_Age:RegionParietal  -4.476e-02  4.477e-02  2.026e+03  -1.000 0.317570
RegionFrontal: Age^2    -1.861e-03  9.406e-04  2.026e+03  -1.978 0.048023 *
RegionOccipital: Age^2   1.622e-04  9.406e-04  2.026e+03   0.172 0.863112
RegionParietal: Age^2    8.377e-04  9.406e-04  2.026e+03   0.891 0.373228
---
Signif. codes:  0 ‘***’ 0.001 ‘**’ 0.01 ‘*’ 0.05 ‘.’ 0.1 ‘ ’ 1
```

Supplementary Table 5b: Aperiodic Exponent and Demographics with Linear Age Effect Only

```
Formula: Exponent ~ EEG_Age * Region + sex + SES + Multilingual + (1 | ID)

Random effects:
 Groups   Name        Variance Std.Dev.
 ID       (Intercept) 0.03474  0.1864
 Residual             0.06315  0.2513
Number of obs: 2220, groups:  ID, 185

Fixed effects:
                          Estimate Std. Error         df t value Pr(>|t|)
(Intercept)              1.509e+00  1.329e-01  2.894e+02  11.357  < 2e-16 ***
EEG_Age                 -1.939e-03  4.927e-03  3.379e+02  -0.394  0.69420
RegionFrontal           -3.000e-02  1.009e-01  2.029e+03  -0.297  0.76626
RegionOccipital          2.762e-01  1.009e-01  2.029e+03   2.738  0.00624 **
RegionParietal           2.082e-01  1.009e-01  2.029e+03   2.064  0.03915 *
sexMale                  2.940e-02  2.981e-02  1.790e+02   0.986  0.32550
SESMiddle                1.069e-01  5.791e-02  1.790e+02   1.845  0.06667 .
SESHigh                  8.068e-02  5.562e-02  1.790e+02   1.451  0.14865
LingualMultiLingual      4.918e-02  4.057e-02  1.790e+02   1.212  0.22712
EEG_Age:RegionFrontal    2.549e-03  4.233e-03  2.029e+03   0.602  0.54716
EEG_Age:RegionOccipital -4.056e-03  4.233e-03  2.029e+03  -0.958  0.33809
EEG_Age:RegionParietal  -5.061e-03  4.233e-03  2.029e+03  -1.196  0.23198
---
Signif. codes:  0 ‘***’ 0.001 ‘**’ 0.01 ‘*’ 0.05 ‘.’ 0.1 ‘ ’ 1
```

Supplementary Table 5c: Aperiodic Exponent and Parent-Rated Language

Formula: Exponent ~ EEG_Age * Region + sex + SES + Multilingual + Parent_English_words * Region + Parent_gestures * Region + Parent_syntax * Region + (1 | ID)

Random effects:

Groups	Name	Variance	Std.Dev.
ID	(Intercept)	0.03736	0.1933
Residual		0.06360	0.2522

Number of obs: 1524, groups: ID, 127

Fixed effects:

	Estimate	Std. Error	df	t value	Pr(>|t|)	
Intercept)	1.518e+00	1.866e-01	2.012e+02	8.132	4.28e-14	***
EEG_Age	-5.425e-03	7.058e-03	2.163e+02	-0.769	0.44295	
RegionFrontal	1.554e-02	1.485e-01	1.382e+03	0.105	0.91668	
RegionOccipital	7.202e-01	1.485e-01	1.382e+03	4.850	1.37e-06	***
RegionParietal	1.906e-01	1.485e-01	1.382e+03	1.283	0.19959	
sexMale	6.297e-02	3.939e-02	1.180e+02	1.599	0.11259	
SESMiddle	1.595e-01	7.587e-02	1.180e+02	2.103	0.03761	*
SESHigh	1.138e-01	7.108e-02	1.180e+02	1.601	0.11202	
LingualMultiLingual	7.571e-02	4.877e-02	1.180e+02	1.552	0.12325	
Parent_English_words	-9.289e-04	1.342e-03	2.108e+02	-0.692	0.48958	
Parent_gestures	2.928e-04	6.050e-03	2.183e+02	0.048	0.96144	
Parent_syntax	4.174e-02	4.219e-02	2.168e+02	0.989	0.32360	
EEG_Age:RegionFrontal	1.536e-03	5.940e-03	1.382e+03	0.259	0.79599	
EEG_Age:RegionOccipital	-1.368e-02	5.940e-03	1.382e+03	-2.303	0.02142	*
EEG_Age:RegionParietal	-6.342e-03	5.940e-03	1.382e+03	-1.068	0.28581	
RegionFrontal:Parent_English_words	-5.059e-04	1.108e-03	1.382e+03	-0.457	0.64796	
RegionOccipital:Parent_English_words	9.397e-04	1.108e-03	1.382e+03	0.848	0.39639	
RegionParietal:Parent_English_words	-6.362e-04	1.108e-03	1.382e+03	-0.574	0.56583	
RegionFrontal:Parent_gestures	-8.817e-04	5.125e-03	1.382e+03	-0.172	0.86342	
RegionOccipital:Parent_gestures	-1.737e-02	5.125e-03	1.382e+03	-3.390	0.00072	***
RegionParietal:Parent_gestures	4.374e-03	5.125e-03	1.382e+03	0.854	0.39352	
RegionFrontal:Parent_syntax	2.370e-02	3.556e-02	1.382e+03	0.667	0.50515	
RegionOccipital:Parent_syntax	3.820e-02	3.556e-02	1.382e+03	1.074	0.28293	
RegionParietal:Parent_syntax	-1.511e-02	3.556e-02	1.382e+03	-0.425	0.67090	

Signif. codes: 0 ‘***’ 0.001 ‘**’ 0.01 ‘*’ 0.05 ‘.’ 0.1 ‘ ’ 1

Supplementary Table 5d: Aperiodic Exponent and Teacher-Rated Language

```
Formula: Exponent ~ EEG_Age * Region + sex + SES + Multilingual + Teacher_English_words * Region +
Teacher_gestures * Region + Teacher_syntax * Region + (1 | ID)

Random effects:
Groups   Name        Variance Std.Dev.
 ID       (Intercept) 0.03324  0.1823
 Residual             0.06158  0.2482
Number of obs: 1620, groups:  ID, 135

Fixed effects:
                                     Estimate Std. Error         df t value Pr(>|t|)
Intercept)                          1.415e+00  1.656e-01  2.093e+02   8.546 2.65e-15 ***
EEG_Age                            -4.174e-03  6.036e-03  2.408e+02  -0.692 0.489897
RegionFrontal                      -5.731e-02  1.288e-01  1.470e+03  -0.445 0.656442
RegionOccipital                     4.542e-01  1.288e-01  1.470e+03   3.526 0.000435 ***
RegionParietal                      2.497e-01  1.288e-01  1.470e+03   1.938 0.052773 .
sexMale                             5.394e-02  3.578e-02  1.260e+02   1.508 0.134134
SESMiddle                           1.511e-01  6.756e-02  1.260e+02   2.236 0.027125 *
SESHigh                             1.256e-01  6.398e-02  1.260e+02   1.964 0.051785 .
LingualMultiLingual                 8.323e-02  4.543e-02  1.260e+02   1.832 0.069270 .
Teacher_English_words              -1.218e-03  1.325e-03  2.432e+02  -0.919 0.358783
Teacher_gestures                    6.615e-03  4.539e-03  2.402e+02   1.457 0.146294
Teacher_syntax                      5.756e-03  4.019e-02  2.389e+02   0.143 0.886239
EEG_Age:RegionFrontal               4.028e-03  5.227e-03  1.470e+03   0.771 0.441067
EEG_Age:RegionOccipital            -7.906e-03  5.227e-03  1.470e+03  -1.513 0.130617
EEG_Age:RegionParietal             -5.786e-03  5.227e-03  1.470e+03  -1.107 0.268537
RegionFrontal:Teacher_English_words   -1.949e-04  1.155e-03  1.470e+03  -0.169 0.865992
RegionOccipital:Teacher_English_words  1.845e-03  1.155e-03  1.470e+03   1.597 0.110403
RegionParietal:Teacher_English_words   6.187e-04  1.155e-03  1.470e+03   0.536 0.592290
RegionFrontal:Teacher_gestures      6.897e-04  3.924e-03  1.470e+03   0.176 0.860511
RegionOccipital:Teacher_gestures   -9.953e-03  3.924e-03  1.470e+03  -2.536 0.011306 *
RegionParietal:Teacher_gestures    -2.804e-03  3.924e-03  1.470e+03  -0.714 0.475091
RegionFrontal:Teacher_syntax       -1.089e-02  3.463e-02  1.470e+03  -0.314 0.753276
RegionOccipital:Teacher_syntax      3.353e-02  3.463e-02  1.470e+03   0.968 0.333130
RegionParietal:Teacher_syntax       1.627e-02  3.463e-02  1.470e+03   0.470 0.638527
---
Signif. codes:  0 ‘***’ 0.001 ‘**’ 0.01 ‘*’ 0.05 ‘.’ 0.1 ‘ ’ 1
```

Supplementary Table 5e: Aperiodic Exponent and Parent-Rated Temperament

Formula: Exponent ~ EEG_Age * Region + sex + SES + Multilingual + Parent_negative_affect * Region + Parent_effortful_control * Region + Parent_surgency * Region + (1 | ID)

Random effects:

Groups	Name	Variance	Std.Dev.
ID	(Intercept)	0.03510	0.1874
Residual		0.06394	0.2529

Number of obs: 2172, groups: ID, 181

Fixed effects:

	Estimate	Std. Error	df	t value	Pr(>|t|)	
(Intercept)	1.433e+00	2.003e-01	2.984e+02	7.153	6.59e-12	***
EEG_Age	-9.887e-04	5.183e-03	3.264e+02	-0.191	0.8488	
RegionFrontal	1.030e-01	1.616e-01	1.976e+03	0.637	0.5239	
RegionOccipital	3.854e-01	1.616e-01	1.976e+03	2.384	0.0172	*
RegionParietal	1.953e-01	1.616e-01	1.976e+03	1.208	0.2270	
sexMale	2.935e-02	3.245e-02	1.720e+02	0.905	0.3669	
SESMiddle	1.017e-01	5.914e-02	1.720e+02	1.719	0.0874	.
SESHigh	7.986e-02	5.699e-02	1.720e+02	1.401	0.1629	
LingualMultiLingual	5.502e-02	4.110e-02	1.720e+02	1.339	0.1824	
Parent_negative_affect	-7.304e-03	2.557e-02	3.283e+02	-0.286	0.7753	
Parent_effortful_control	2.093e-03	2.494e-02	3.220e+02	0.084	0.9332	
Parent_surgency	1.375e-02	2.876e-02	3.069e+02	0.478	0.6330	
EEG_Age:RegionFrontal	2.392e-03	4.468e-03	1.976e+03	0.535	0.5925	
EEG_Age:RegionOccipital	-5.314e-03	4.468e-03	1.976e+03	-1.189	0.2344	
EEG_Age:RegionParietal	-6.366e-03	4.468e-03	1.976e+03	-1.425	0.1544	
RegionFrontal:Parent_negative_affect	3.348e-03	2.213e-02	1.976e+03	0.151	0.8798	
RegionOccipital:Parent_negative_affect	2.326e-03	2.213e-02	1.976e+03	0.105	0.9163	
RegionParietal:Parent_negative_affect	-1.125e-02	2.213e-02	1.976e+03	-0.508	0.6113	
RegionFrontal:Parent_effortful_control	-1.444e-02	2.130e-02	1.976e+03	-0.678	0.4980	
RegionOccipital:Parent_effortful_control	7.127e-03	2.130e-02	1.976e+03	0.335	0.7380	
RegionParietal:Parent_effortful_control	7.893e-03	2.130e-02	1.976e+03	0.371	0.7110	
RegionFrontal:Parent_surgency	-2.016e-02	2.372e-02	1.976e+03	-0.850	0.3955	
RegionOccipital:Parent_surgency	-2.536e-02	2.372e-02	1.976e+03	-1.069	0.2852	
RegionParietal:Parent_surgency	8.965e-03	2.372e-02	1.976e+03	0.378	0.7055	

Signif. codes: 0 '***' 0.001 '**' 0.01 '*' 0.05 '.' 0.1 ' ' 1

Supplementary Table 5f: Aperiodic Exponent and Teacher-Rated Temperament

Formula: Exponent ~ EEG_Age * Region + sex + SES + Multilingual + Teacher_negative_affect * Region + Teacher_effortful_control * Region + Teacher_surgency * Region + (1 | ID)

Random effects:

Groups	Name	Variance	Std.Dev.
ID	(Intercept)	0.03690	0.1921
Residual		0.06385	0.2527

Number of obs: 1992, groups: ID, 166

Fixed effects:

	Estimate	Std. Error	df	t value	Pr(>|t|)	
(Intercept)	1.364e+00	1.947e-01	2.644e+02	7.007	2.02e-11	***
EEG_Age	-3.494e-04	5.499e-03	2.871e+02	-0.064	0.9494	
RegionFrontal	7.314e-02	1.533e-01	1.811e+03	0.477	0.6333	
RegionOccipital	3.596e-01	1.533e-01	1.811e+03	2.346	0.0191	*
RegionParietal	2.435e-01	1.533e-01	1.811e+03	1.588	0.1124	
sexMale	2.466e-02	3.297e-02	1.570e+02	0.748	0.4556	
SESMiddle	1.378e-01	6.298e-02	1.570e+02	2.188	0.0301	*
SESHigh	1.057e-01	6.010e-02	1.570e+02	1.758	0.0806	.
MultiLingual	5.723e-02	4.425e-02	1.570e+02	1.293	0.1978	
Teacher_negative_affect	-7.236e-03	2.468e-02	2.881e+02	-0.293	0.7696	
Teacher_effortful_control	1.091e-02	2.602e-02	2.893e+02	0.419	0.6755	
Teacher_surgency	1.735e-02	1.706e-02	2.927e+02	1.017	0.3099	
EEG_Age:RegionFrontal	3.669e-03	4.620e-03	1.811e+03	0.794	0.4272	
EEG_Age:RegionOccipital	-2.923e-03	4.620e-03	1.811e+03	-0.633	0.5269	
EEG_Age:RegionParietal	-4.814e-03	4.620e-03	1.811e+03	-1.042	0.2975	
RegionFrontal:Teacher_negative_affect	-3.632e-02	2.079e-02	1.811e+03	-1.747	0.0808	.
RegionOccipital:Teacher_negative_affect	1.892e-02	2.079e-02	1.811e+03	0.910	0.3629	
RegionParietal:Teacher_negative_affect	3.503e-06	2.079e-02	1.811e+03	0.000	0.9999	
RegionFrontal:Teacher_effortful_control	-1.615e-02	2.198e-02	1.811e+03	-0.735	0.4627	
RegionOccipital:Teacher_effortful_control	-2.188e-02	2.198e-02	1.811e+03	-0.995	0.3197	
RegionParietal:Teacher_effortful_control	-8.450e-03	2.198e-02	1.811e+03	-0.384	0.7007	
RegionFrontal:Teacher_surgency	-3.568e-03	1.453e-02	1.811e+03	-0.246	0.8060	
RegionOccipital:Teacher_surgency	-1.995e-02	1.453e-02	1.811e+03	-1.373	0.1699	
RegionParietal:Teacher_surgency	-4.477e-03	1.453e-02	1.811e+03	-0.308	0.7580	

Signif. codes: 0 ‘***’ 0.001 ‘**’ 0.01 ‘*’ 0.05 ‘.’ 0.1 ‘ ’ 1
